# Trends and all-cause mortality associated with multimorbidity of non-communicable diseases among adults in the United States, 1999-2018: a retrospective cohort study

**DOI:** 10.4178/epih.e2023023

**Published:** 2023-02-14

**Authors:** Mengzi Sun, Ling Wang, Xuhan Wang, Li Tong, Lina Jin, Bo Li

**Affiliations:** Department of Epidemiology and Biostatistics, School of Public Health, Jilin University, Changchun, China

**Keywords:** Multimorbidity, Noncommunicable diseases, Mortality, National Health and Nutrition Examination Survey, Trends

## Abstract

**OBJECTIVES:**

Multimorbidity of non-communicable diseases (NCDs) has brought enormous challenges to public health, becoming a major medical burden. However, the patterns, temporal trends, and all-cause mortality associated with NCD multimorbidity over time have not been well described in the United States.

**METHODS:**

All adult participants were sourced from nationally representative data from the National Health and Nutrition Examination Survey. In total, 55,081 participants were included in trend analysis, and 38,977 participants were included in Cox regression.

**RESULTS:**

The 5 NCDs with the largest increases over the study period were diabetes, osteoporosis, obesity, liver conditions, and cancer. The estimated prevalence of multimorbidity increased with age, especially for middle-aged participants with 5 or more NCDs; in general, the prevalence of NCD multimorbidity was higher among females than males. Participants with 5 or more NCDs were at 4.49 times the risk of all-cause mortality of participants without any diseases. Significant interactions were found between multimorbidity and age group (p for interaction <0.001), race/ethnicity (p for interaction<0.001), and educational attainment (p for interaction=0.010).

**CONCLUSIONS:**

The prevalence of multiple NCDs significantly increased from 1999 to 2018. Those with 5 or more NCDs had the highest risk of all-cause mortality, especially among the young population. The data reported by this study could serve as a reference for additional NCD research.

## INTRODUCTION

Multimorbidity is the co-occurrence of 2 or more diseases [1], in which no single condition holds priority over any of the co-occurring conditions [2]. Multimorbidity of non-communicable diseases (NCDs) is extremely common and is considered an enormous challenge in public health, constituting a major medical burden since the last century [3]. Previous studies have indicated that the prevalence of multimorbidity is growing along with the increase in life expectancy and global population aging [4,5]. A separate retrospective observational study in the United Kingdom reported that 20% of patients had at least 2 chronic conditions [6].

Additionally, a systematic analysis for the Global Burden of Disease Study in 1990-2013 indicated that the multimorbidity of NCDs becomes substantially more frequent with age [7]; another study similarly showed that the prevalence of NCD multimorbidity increases with age among United States adults [8]. Furthermore, most deaths in developed countries are thought to occur in people over 65 years old with multiple chronic conditions causing or contributing to death [9]. Due to the complexity of NCD multimorbidity, it is essential to examine associated patterns utilizing nationally representative data. In addition, socio-demographic correlates of NCD multimorbidity have not yet been systematically evaluated in the population.

For these reasons, we examined the trends and all-cause mortality associated with multimorbidity of NCDs in United States adults, both overall and according to socio-demographic characteristics, utilizing data from the National Health and Nutrition Examination Survey (NHANES). The results of this study could inform additional NCD research.

## MATERIALS AND METHODS

### Study population

The NHANES, a population-based survey designed to collect information on the health and nutrition of United States households, has included a nationally representative, complex, stratified, multistage probability sample of the civilian non-institutionalized United States population in continuous 2-year cycles since 1999 [10].

The present study included 3 age groups: young adults (20-39 years old), middle-aged adults (40-64 years old), and older adults (≥ 65 years old). Information on socio-demographic characteristics, weight, and 48 diseases were combined into a single dataset for each data cycle from 1999-2000 to 2017-2018. The trend of NCD multimorbidity is reflected in the prevalence of 2 or more diseases in adults over the age of 20 years from 1999-2000 to 2017-2018. The trends of each NCD were examined by age (20-39, 40-64, and ≥ 65 years) and sex (male and female). Data on all-cause mortality were obtained using a probabilistic match between NHANES and the National Death Index death certificate records.

This study had 2 parts. First, in a serial cross-sectional study, we included a total of 55,081 participants from 1999-2018 for the trend analysis. Second, in a retrospective study, 38,977 participants from 1999-2018 were included in the all-cause mortality analysis due to missing covariates. The flowchart of participant inclusion is shown in Supplementary Material 1.

### Disease definitions

For the NHANES (1999-2018), NCDs were diagnosed based on several questions. Diabetes was defined as meeting any of the following conditions: ever having been diagnosed with diabetes by a doctor; currently taking insulin; ever having taken diabetes pills to lower blood sugar; having a fasting glucose concentration of at least 126 mg/dL; or having a hemoglobin A1c level of at least 6.5% [11,12]. Hypertension was defined as meeting any of the following conditions: ever having been diagnosed with high blood pressure by a doctor; ever having been told to take prescription medication for hypertension; currently taking prescription medicine for hypertension; or exhibiting an average systolic blood pressure greater than 130 mmHg or a diastolic blood pressure greater than 80 mmHg in the NHANES examination section [13]. Hyperlipidemia was defined as meeting any of the following conditions: ever having been informed of having a high cholesterol level by a doctor; ever having been told to take a prescription for cholesterol; currently taking prescription medicine for cholesterol; or having a low-density lipoprotein level of at least 190 mg/mL [14]. Obesity was defined as a body mass index greater than 30 kg/m^2^ [2,15].

The following NCDs were identified based on the question “Have you ever been told that you have this disease by a doctor?”: asthma, arthritis, congestive heart failure, coronary heart disease, heart attack, stroke, emphysema, thyroid problem, psoriasis, gout, chronic obstructive pulmonary disease, gout, weak or failing kidneys, kidney stones, osteoporosis/brittle bones, and chronic bronchitis. Cancers were identified based on the questions “Has a doctor ever told you that you had cancer or malignancy?” and, if so, “What kind of cancer?”. The cancers included bladder cancer, blood cancer, bone cancer, brain cancer, breast cancer, cervical cancer, colon cancer, esophageal cancer, gallbladder cancer, kidney cancer, larynx/windpipe cancer, leukemia, liver cancer, lung cancer, lymphoma/Hodgkin lymphoma, melanoma, mouth/tongue/lip cancer, nervous system cancer, ovarian cancer, pancreatic cancer, prostate cancer, rectal cancer, non-melanoma skin cancer, unknown skin cancer, soft tissue cancer, stomach cancer, testicular cancer, thyroid cancer, uterine cancer, and any other type of cancer.

### Assessment of socio-demographic and lifestyle characteristics

Self-reported socio-demographic characteristics included age, sex, race/ethnicity (Mexican American, other Hispanic, non-Hispanic White, non-Hispanic Black, or other race), annual household income (< US$25,000, 25,000-75,000, or ≥ 75,000), educational attainment (less than high school, high school, or more than high school), physical activity (never, vigorous, or moderate), and marriage status (live together or single). Smoking status was divided into 3 categories. Non-smokers were defined as those who had smoked fewer than 100 cigarettes in their lifetime, former smokers were defined as those who had smoked at least 100 cigarettes but did not smoke currently, and current smokers were defined as participants who had smoked at least 100 cigarettes and reported the number of cigarettes smoked per day in the past 30 days [16]. Drinking status was similarly divided into 3 categories; non-drinkers were defined as participants who had consumed fewer than 12 alcohol-based drinks in their lifetime, former drinkers were defined as participants who had consumed at least 12 drinks in their lifetime but not in the past year, and current drinkers were defined as participants who had consumed at least 12 drinks in the past year. Current drinkers reported the number of drinks consumed per week [16]. Weight was measured during the physical examination at the mobile examination center, and body mass index was calculated as weight in kilograms divided by height in meters squared. The survey had 2 parts: a home interview and a health examination. During the in-home interview, participants were asked questions about health status and disease history. The examination component consisted of medical tests, including blood pressure measurement, and examinations administered by highly trained medical personnel [17].

### Statistical analysis

For all analyses, we used NHANES interview sample weights, stratification, and clustering of the complex sampling design adjusted with non-response, non-coverage, and unequal probabilities of selection. All estimates were weighted to be nationally representative [10]. We categorized NCDs in the following format: S[χ]=[i | participant i suffers from×NCD(s)]. That is, S[0] is the subpopulation with no NCDs; S[1] is the subpopulation with only 1 NCD; S[2-4] is the subpopulation with 2 to 4 NCDs; and S[5+] is the subpopulation with 5 or more NCDs. The number and weighted prevalence of S[0], S[1], S[2-4], and S[5+] were calculated by age, sex, race/ethnicity, annual household income, educational attainment, physical activity, and marriage status for each 2-year NHANES cycle between 1999 and 2018.

Estimates of the crude weighted prevalence rates of the S[0], S[1], S[2-4], and S[5+] subpopulations were calculated by NHANES cycle among all adults, with and without cancer. The p for trend was estimated using the survey cycle as a continuous variable and survey-weighted logistic regression. The ratio of prevalence was estimated using the relative risk in complex samples via SPSS (IBM Corp., Armonk, NY, USA).

The statistical significance of trends was assessed at the 2-sided α= 0.05 level. In the Results section, an increase refers to a p for trend< 0.05 and a ratio> 1, a decrease refers to a p for trend< 0.05 and a ratio < 1, and a stable finding refers to a p for trend ≥ 0.05.

To determine the risk of all-cause mortality associated with each number of NCDs, we created Cox proportional hazards models while adjusting for age, sex, race/ethnicity, educational attainment, annual household income, physical activity, marriage status, smoking status, and drinking status. Although S[2+] represents multimorbidity, separate S[2-4] and S[5+] categories were examined for further survival analysis, which could then be used to explore the impact of the number of multimorbidities on all-caused death. Hazard ratios (HRs) with 95% confidence intervals (CIs) were estimated using univariate and multivariable Cox proportional hazards models with time in the study as the underlying time metric for calculation. Sensitive analysis of Cox regression was conducted via stratified analysis, and the p-interaction between multimorbidity and each stratified variable was also tested. All statistical tests were 2-sided, and p-values of < 0.05 were considered to indicate statistical significance. Data were analyzed using SPSS version 26.0 (IBM Corp., Armonk, NY, USA).

### Ethics statement

The NHANES protocols were approved by the Institutional Review Board of the National Center for Health Statistics of the Centers for Disease Control and Prevention (NCHS IRB/ERB Protocol #98-12, Protocol #2005-06, Protocol #2011-17, Protocol #2018-01). Written informed consent was obtained from each participant before participation in this study.

## RESULTS

In total, 101,316 individuals participated in the 10 survey cycles (1999-2018). After excluding individuals under the age of 20 years, 55,081 adults were included in this study. The sample size per cycle ranged from 4,880 to 6,218 participants, from 1999-2000 to 2017-2018. Unweighted sample sizes in the 2017-2018 cycle by socio-demographic characteristics are presented in Table 1, and the 1999-2000 to 2015-2016 cycles are illustrated in the Supplementary Materials 2-10.

In the 2017-2018 cycle, a substantial proportion of the population had multimorbidity. The estimated prevalence rates of S[2-4] and S[5+] among adults were 40.8% and 18.1%, respectively. Most of the participants without multimorbidity were young adults. Adults aged 40 years to 64 years old had the highest proportion of S[2-4], while older adults had the highest proportion of S[5+]. The estimated prevalence of multimorbidity across cycles ranged from 33.8% to 40.9% for S[2-4] and 5.7% to 18.1% for S[5+] (Supplementary Materials 2-10).

Relative to the 1999-2000 cycle, a significant increase was noted in the 2017-2018 estimated prevalence of multimorbidity (p for trend < 0.001), especially for S[5+]. In contrast, the estimated prevalence rates of S[0] and S[1] showed statistically significant declines from 1999 onward (p for trend < 0.001). Throughout all 10 cycles, a substantial proportion of the population experienced 2 to 4 NCDs (33.8 to 40.9%; p for trend < 0.001); additionally, although a minority of individuals reported having 5 or more NCDs, the estimated prevalence of the S[5+] condition increased significantly over time (5.7 to 18.1%, p for trend < 0.001; Table 2). The estimated prevalence of NCD multimorbidity was lower among the participants without cancer than among all adults.

The estimated prevalence rate of each NCD and cancer among all adults in 1999-2018 is shown in Supplementary Material 11. Among the 20 NCDs, the 5 most prevalent were hypertension (35.2%), obesity (33.4%), hyperlipidemia (31.4%), arthritis (24.4%), and asthma (13.9%). The prevalence of most NCDs increased over the 20 years observed (p for trend < 0.05), and the 5 with the largest increases were diabetes, osteoporosis, obesity, liver conditions, and cancer. The estimated prevalence of each cancer is also shown in Supplementary Material 12.

The most common 10 pairs of multimorbid NCDs were almost identical throughout all 10 cycles and included hypertension, hyperlipidemia, obesity, arthritis, and diabetes. In the 2013-2014 cycle, the estimated prevalence of hypertension and hyperlipidemia (22.0%) was the highest of these pairs, and a significant upward trend was noted after 1999 (p for trend < 0.001). The estimated prevalence of the top 10 pairs of multimorbidities significantly increased throughout the 10 cycles (p for trend < 0.001; Table 3).

Next, we divided participants into age and sex subgroups to describe the estimated prevalence of each NCD, as shown in Supplementary Material 13. Except for asthma, all NCDs differed significantly in prevalence among age subgroups. Obesity had the highest estimated prevalence in the middle-aged group, while the other conditions were most prevalent among older adults. Compared with females, males had a higher estimated prevalence of congestive heart failure, coronary heart disease, heart attack, gout, hypertension, hyperlipidemia, and angina and a lower estimated prevalence of asthma, arthritis, thyroid problems, osteoporosis, chronic bronchitis, and cancers (p< 0.05).

**Figure 1.**
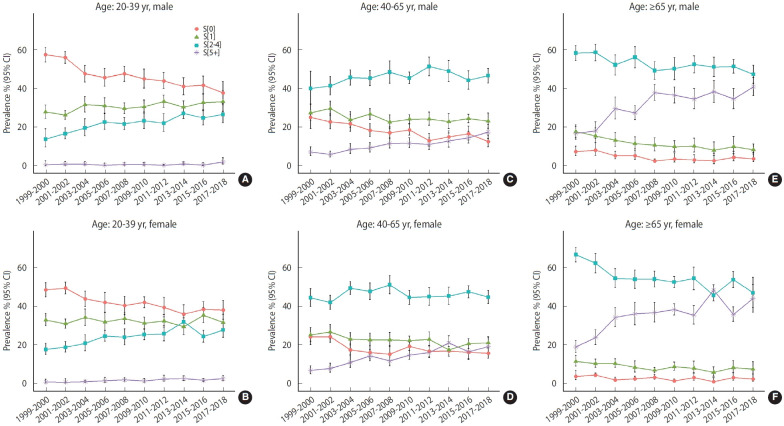
The estimated prevalence of category of NCDs (S[0], S[1], S[2-4], S[5+]) by different age group and sex group. (A) for males of 20-39 years old; (B) for females of 20-39 years old; (C) for males of 40-65 years old; (D) for females of 40-65 years old; (E) for males of ≥65 years old; (F) for females of ≥65 years old. CI, confidence interval.

Figure 1 shows the estimated prevalence of number of NCDs by age and sex groups. In general, the estimated prevalence of S[0] declined, and the estimated prevalence of multimorbidity significantly increased. The estimated prevalence of multimorbidity was lower than that of S[0] in young adults, but the estimated prevalence of multimorbidity was higher in adults aged over 40 years, especially for those aged 65 years or more. The estimated prevalence of multimorbidity increased with age, and the trends of S[5+] exhibited an obvious acceleration from middle age onward. Compared with middle-aged adults, the older population exhibited an even higher estimated prevalence of multimorbidity, particularly among females.

We next explored the all-cause mortality of patients with NCD multimorbidity using Cox proportional hazards models based on participants from 1999-2018, as shown in Table 4. Survival curves by number of NCDs are shown in Supplementary Material 14. Compared with the population with no disease, participants in the S[5+] category had 4.49 times the risk of all-cause mortality after adjustment. We then explored the interaction between each variable and multimorbidity. As shown in Table 5, interactions were found between multimorbidity and age group (p for interaction< 0.001), race/ethnicity (p for interaction< 0.001), and educational attainment (p for interaction= 0.010). The HRs of all-cause mortality increased with the number of multimorbidities. Notably, within the S[5+] subcategory, the HR was highest among the young population (HR, 17.85; 95% CI, 8.33 to 38.28) and lowest among the elderly (HR, 2.10; 95% CI, 1.59 to 2.78). Among the subgroups categorized by race/ethnicity, the “other Hispanic” category displayed the highest HRs. Regarding educational attainment, both the groups with lower-than-high-school and higher-than-high-school education levels had higher HRs of all-cause mortality than the group with a high school education.

## DISCUSSION

In this nationally representative sample of the adult population in the United States, the estimated prevalence of multimorbidity was consistently high and significantly increased from 1999 to 2018, especially for the middle-aged and elderly. Furthermore, the estimated prevalence of ≥ 5 multimorbidities accelerated starting in middle age. Last but most importantly, participants with 5 or more NCDs had the highest risk of all-cause mortality relative to participants without any diseases, especially among the young population. To our knowledge, this was the first study of the prevalence of multimorbidity and all-cause mortality associated with NCDs in the United States, and the data reported in this study could inform additional NCD research.

Until now, the prevention and treatment of NCDs in the population have primarily been focused on single NCDs rather than the multimorbidity of these diseases. However, the prevention of multimorbidity cannot simply be viewed as an accumulation of many instances of single NCD prevention, but rather as a more effective and easier overall strategy to implement. Furthermore, populations with NCD multimorbidity are at an increased risk of mortality, morbidity, hospitalization, high medical costs, and adverse events [18], and this particularly heavy additional burden may be imposed by multimorbidity [19,20]. Our study revealed that the percentage of the population experiencing a single NCD declined over time, whereas the prevalence of NCD multimorbidity was high and trended upward. Therefore, the importance of multimorbidity prevention is obvious. In particular, understanding the situation and trends in the multimorbidity of NCDs is a critical step before population-wide strategies to prevent NCD multimorbidity can be developed and implemented.

The prevalence of multimorbidity is high not only in the United States, but also in other countries. A large United Kingdombased study found that 42% of the population had at least 1 NCD and 23% had multimorbidity, with two-thirds of people aged 65 years or over having multimorbidity [21]. Among Canadians aged 40 years, the reported prevalence rates of 2 or more and 3 or more chronic conditions were 26.5% and 10.2%, respectively [22]. Another previous study revealed that NCD multimorbidity had a monotonic increasing trend from 2003 to 2013 [23]. A study on United States adults reported that the prevalence of multiple chronic conditions increased with age [8], which was consistent with our study. These increasing trends suggest that we should focus on the health of the entire population with regard to multimorbidity. Moreover, with the growing number of elderly people in the world, the prevention of multimorbidity has become an urgent and serious problem.

Interestingly, the 10 most common pairs of multimorbid NCDs were almost identical between 1999 and 2016, suggesting that the morbidities may have potential mechanisms or risk factors in common. A study among older adults in Florida and the United States as a whole showed that the most prevalent pairs of chronic conditions were hyperlipidemia and hypertension, hypertension and ischemic heart disease, and diabetes and hypertension [23], which aligned with our findings. Another study reported that 73% of hypertensive patients presented with dyslipidemia [24], perhaps because hypercholesterolemia may increase sensitivity to some of the mechanisms involved in blood pressure elevation [25]. The prevalence of obesity has risen globally over the past 4 decades [26], and the associations of obesity with hypertension and hyperlipidemia have been widely explored [27,28]. Moreover, arthritis co-occurred with hypertension, hyperlipidemia, obesity, and diabetes, potentially because the promoted systemic cardiovascular risk factors are produced in locally affected joints through proinflammatory cytokines (including dyslipidemia and oxidative stress) [29,30]. Additionally, individuals with one of these NCDs should pay special attention to preventing other NCDs; that is, multimorbidity studies can also provide effective preventive strategies. Therefore, relative to studies of a single NCD, studies of multimorbidity may provide a novel and creative rationale to better understand the mechanisms and prevention of NCDs, since potential common mechanisms or risk factors would be highly enhanced in multimorbidity.

Furthermore, we observed an apparent acceleration in the prevalence of 5 or more NCDs beginning in middle age. This indicates that middle-aged individuals form the key population for multimorbidity prevention. Although a consensus may already exist that the middle-aged and elderly tend to experience the most NCDs, we identified and quantified the specific level of risk. Based on our findings, interventions to prevent multimorbidity should be enacted earlier, in middle age, rather than in the elderly. Other-wise, the multimorbidity of this subpopulation would contribute a heavy burden to society in the future, including an increasing risk of all-cause mortality.

Notably, the all-cause mortality associated with at least 5 NCDs was highest in the young population, rather than the elderly, as one may assume. Additionally, we identified interactions of the number of NCDs with both race and educational attainment. The patterns of NCDs clearly differed among races. Regarding education, one study indicated that all-cause mortality rates were approximately 2 times higher among adults with the least education than among those with the most [31]. Another study indicated that veterans’ mortality rates varied substantially by racial and ethnic group [32]. However, the “other Hispanic” and “higher than high school” populations had the highest risks of all-cause mortality associated with multimorbidity. Therefore, intervention strategies and measures should be applied to these key populations before the situation deteriorates, and the prevention of multimorbidity should be implemented at an age prior to the most seriously impacted age group. Among patients with multimorbidity, the population with 5 or more NCDs requires special attention; furthermore, within that population, young people should be a particular focus.

This study has several limitations. First, self-reported health conditions may not reflect the true number of NCDs. However, self-report bias was unlikely to affect the observed secular trends over time. Second, the prevalence was not measured over the entire year, but rather was based on the pre-existence of the condition prior to the date of the investigation. Third, participants who re-fused to answer or were not familiar with the conditions and thus had missing values were regarded as not having the NCDs, which could result in underestimation of prevalence. Overall, measuring and understanding the prevalence of multimorbidity is both critical and urgent for the early prevention of NCDs.

In conclusion, the estimated prevalence of NCD multimorbidity was consist-ently high and displayed a significant upward trend from 1999 through 2018, especially for those aged over 40 years with 5 or more NCDs, and this prevalence accelerated beginning in middle age. Meanwhile, the 10 most common pairs of multimorbid conditions were nearly identical between 1999 and 2018, and the estimated prevalence rates of these pairs also appeared to be increasing. The number of NCDs and the age of participants were associated with all-cause mortality. Populations with 5 or more NCDs had the highest risk of all-cause mortality, especially young adults.

## DATA AVAILABILITY

The data described in the manuscript, code book, and analytic code will be made publicly and freely available without restriction at https://www.cdc.gov/nchs/nhanes.

## Figures and Tables

**Table 1.** Sample sizes regarding multimorbidity of NCDs among adults in the United States by socio-demographic characteristics, NHANES 2017-2018^1,2^

Characteristics		No. of participants by no. of NCDs				
		Total	S[0]	S[1]	S[2-4]	S[5+]
Overall		5,569 (100.0)	954 (19.2)	1,129 (21.9)	2,301 (40.8)	1,185 (18.1)
Age (yr)						
	20-39	1,687 (36.1)	626 (69.8)	549 (51.5)	469 (25.9)	43 (4.9)
	40-64	2,382 (43.6)	284 (27.6)	470 (41.5)	1,153 (51.1)	475 (46.0)
	≥65	1,500 (20.3)	44 (2.6)	110 (7.0)	679 (23.0)	667 (49.1)
Sex						
	Male	2,702 (48.1)	472 (47.5)	528 (48.2)	1,148 (50.2)	554 (44.1)
	Female	2,867 (51.9)	482 (52.5)	601 (51.8)	1,153 (49.8)	631 (55.9)
Race/ethnicity						
	Mexican American	735 (8.8)	151 (11.3)	172 (10.9)	304 (8.3)	108 (4.8)
	Other Hispanic	517 (6.9)	103 (9.6)	92 (6.8)	228 (6.6)	94 (5.0)
	Non-Hispanic White	1,935 (62.3)	253 (55.2)	330 (57.9)	802 (64.0)	550 (71.4)
	Non-Hispanic Black	1,298 (11.5)	199 (11.9)	258 (11.5)	553 (11.5)	288 (11.0)
	Other race	1,084 (10.4)	248 (12.0)	277 (12.9)	414 (9.6)	145 (7.7)
Annual household income (US$)						
	<25,000	1,264 (16.8)	190 (16.5)	214 (13.5)	486 (15.8)	374 (23.6)
	25,000-75,000	2,287 (42.0)	363 (41.1)	472 (43.5)	960 (40.0)	492 (45.6)
	≥75,000	1,436 (41.2)	281 (42.5)	323 (43.1)	628 (44.2)	204 (30.8)
Educational attainment						
	Less than high school	1,117 (11.3)	173 (10.6)	225 (11.6)	443 (10.3)	276 (14.1)
	High school	1,325 (27.2)	219 (25.7)	238 (23.8)	564 (27.9)	304 (31.1)
	More than high school	3,114 (61.5)	560 (63.7)	662 (64.6)	1,291 (61.8)	601 (54.8)
Marital status						
	Living together	3,252 (62.0)	555 (56.5)	663 (61.9)	1,403 (64.8)	631 (61.7)
	Single	2,311 (38.0)	398 (43.5)	465 (38.1)	897 (35.2)	551 (38.3)
Physical activity						
	Never	3,030 (47.9)	509 (48.8)	614 (47.7)	1,200 (44.6)	707 (54.6)
	Vigorous	223 (4.1)	41 (4.0)	46 (2.8)	100 (5.3)	36 (2.9)
	Moderate	2,316 (48.0)	404 (47.3)	469 (49.5)	1,001 (50.1)	442 (42.4)
Smoking status						
	Never	3,234 (57.7)	652 (66.9)	755 (62.9)	1,316 (57.0)	511 (43.3)
	Current	1,006 (17.4)	169 (17.0)	202 (18.2)	434 (17.2)	201 (17.3)
	Former	1,329 (24.9)	133 (16.1)	172 (18.9)	551 (25.8)	473 (39.4)
Drinking status						
	Never	509 (7.4)	96 (8.2)	118 (8.6)	213 (7.2)	82 (5.3)
	Current	3,337 (75.9)	575 (81.2)	743 (81.4)	1,443 (76.6)	576 (62.3)
	Former	1,034 (16.8)	99 (10.6)	133 (9.9)	408 (16.2)	394 (32.3)

Values are presented as number (weighted %). NCDs, non-communicable diseases; NHANES, National Health and Nutrition Examination Survey.

1 Participant characteristics are presented by number of NCDs: S[0] (no NCDs); S[1] (1 NCD), S[2-4] (2 to 4 NCDs), and S[5+] (5 or more NCDs); Sample size was weighted to be nationally representative.

2 Number of participants within each group may not sum to the overall value due to missing data; Similarly, weighted percentages may not sum to 100% due to missing data.

**Table 2.** Crude weighted trends in multimorbidity of NCDs among adults in the United States, NHANES 1999-2018

No. of NCDs		Total	Trends in multimorbidity of NCDs in NHANES cycle years										Ratio of prevalence: 2017-2018 vs. 1999-2000	p-value	p-trend
			1999-2000 (n=4,880)	2001-2002 (n=5,411)	2003-2004 (n=5,041)	2005-2006 (n=4,979)	2007-2008 (n=5,935)	2009-2010 (n=6,218)	2011-2012 (n=5,560)	2013-2014 (n=5,769)	2015-2016 (n=5,719)	2017-2018 (n=5,569)			
Total (n=55,081)															
	S[0]	12,485 (24.7)	1,360 (33.8)	1,525 (31.5)	1,186 (26.8)	1,250 (24.9)	1,254 (24.1)	1,377 (25.1)	1,238 (22.7)	1,190 (21.5)	1,151 (20.7)	954 (19.2)	0.677	<0.001	<0.001
	S[1]	12,412 (23.9)	1,247 (26.7)	1,315 (26.6)	1,181 (25.5)	1,192 (24.7)	1,253 (23.1)	1,365 (23.3)	1,234 (23.9)	1,233 (21.1)	1,263 (23.2)	1,129 (21.9)	0.870	<0.001	<0.001
	S[2-4]	21,989 (38.7)	1,910 (33.8)	2,051 (34.4)	2,038 (37.6)	1,937 (39.3)	2,410 (39.9)	2,487 (38.8)	2,251 (40.4)	2,329 (40.9)	2,275 (39.7)	2,301 (40.8)	1.185	<0.001	<0.001
	S[5+]	8,195 (12.7)	363 (5.7)	520 (7.6)	636 (10.1)	600 (11.1)	1,018 (12.9)	989 (12.9)	837 (12.9)	1,017 (16.5)	1,030 (16.3)	1,185 (18.1)	2.380	<0.001	<0.001
Without cancer (n=49,915)															
	S[0]	12,485 (27.3)	1,360 (36.3)	1,525 (34.3)	1,186 (29.3)	1,250 (27.1)	1,254 (26.5)	1,377 (27.8)	1,238 (25.2)	1,190 (24.2)	1,151 (23.3)	954 (21.6)	0.694	<0.001	<0.001
	S[1]	12,074 (25.5)	1,198 (27.3)	1,276 (28.2)	1,149 (27.1)	1,167 (26.4)	1,223 (24.7)	1,318 (24.7)	1,211 (25.9)	1,200 (22.7)	1,237 (25.4)	1,095 (23.6)	0.902	0.002	0.001
	S[2-4]	19,546 (37.6)	1,672 (32.0)	1,749 (31.9)	1,789 (36.0)	1,730 (38.1)	2,151 (39.0)	2,211 (37.9)	2,016 (39.1)	2,106 (40.7)	2,046 (39.2)	2,076 (40.7)	1.232	<0.001	<0.001
	S[5+]	5,810 (9.5)	264 (4.4)	341 (5.5)	439 (7.6)	418 (8.4)	731 (9.8)	692 (9.6)	607 (9.8)	726 (12.4)	736 (12.1)	856 (14.1)	2.315	<0.001	<0.001

Values are presented as number (weighted %). NCDs, non-communicable diseases; NHANES, National Health and Nutrition Examination Survey.

**Table 3.** Crude weighted trends in the 10 most common pairs of multimorbid NCDs among adults in the United States, NHANES 1999-2018

NCD pair	Trends in NCD multimorbidity in NHANES cycle years										Ratio of prevalence: 2017-2018 vs. 1999-2000	p-value	p-trend
	1999-2000	2001-2002	2003-2004	2005-2006	2007-2008	2009-2010	2011-2012	2013-2014	2015-2016	2017-2018			
Hypertension and hyperlipidemia	661 (12.0)	749 (11.9)	907 (16.1)	815 (15.7)	1,179 (16.8)	1,184 (16.6)	1,245 (20.0)	1,357 (22.0)	1,349 (20.8)	1,398 (21.0)	1.503	<0.001	<0.001
Hypertension and obesity	716 (12.5)	671 (11.8)	777 (14.2)	776 (14.9)	1,071 (15.1)	1,197 (17.5)	1,009 (16.4)	1,108 (18.7)	1,141 (18.9)	1,196 (20.3)	1.415	<0.001	<0.001
Arthritis and hypertension	677 (10.3)	741 (10.6)	843 (13.4)	736 (13.4)	1,079 (14.4)	1,048 (13.4)	902 (13.9)	985 (16.5)	943 (15.6)	1,118 (16.8)	1.410	<0.001	<0.001
Arthritis and hyperlipidemia	467 (8.1)	540 (8.3)	653 (11.2)	563 (11.3)	852 (12.0)	805 (11.2)	786 (12.4)	902 (16.0)	863 (15.3)	1,000 (15.6)	1.582	<0.001	<0.001
Hyperlipidemia and obesity	402 (8.1)	442 (7.8)	540 (10.8)	566 (12.2)	771 (11.4)	837 (12.6)	798 (13.4)	922 (16.5)	933 (16.2)	903 (15.4)	1.574	<0.001	<0.001
Arthritis and obesity	438 (7.6)	412 (7.1)	512 (9.2)	533 (10.5)	749 (10.7)	765 (10.8)	608 (9.7)	723 (12.9)	734 (13.0)	805 (13.6)	1.495	<0.001	<0.001
Hypertension and diabetes	391 (4.7)	431 (5.5)	479 (7.1)	439 (6.7)	730 (8.2)	735 (8.4)	667 (8.5)	667 (9.1)	728 (9.5)	799 (10.6)	1.750	<0.001	<0.001
Hyperlipidemia and diabetes	244 (3.3)	295 (3.9)	362 (5.9)	319 (5.3)	559 (6.6)	563 (6.7)	639 (8.2)	656 (9.2)	695 (9.2)	751 (9.8)	2.149	<0.001	<0.001
Diabetes and obesity	271 (3.9)	279 (4.0)	327 (5.3)	341 (5.5)	566 (7.2)	601 (7.6)	512 (7.3)	517 (7.8)	588 (8.4)	636 (9.2)	1.814	<0.001	<0.001
Arthritis and diabetes	287 (3.6)	265 (3.4)	316 (4.6)	306 (4.6)	487 (5.5)	475 (5.4)	412 (5.0)	407 (5.8)	442 (6.3)	560 (7.1)	1.561	<0.001	<0.001

Values are presented as number (weighted %). NCD, non-communicable disease; NHANES, National Health and Nutrition Examination Survey.

**Table 4.** Cox proportional hazards models^1^ of the associations between non-communicable disease (NCD) multimorbidity and all-cause mortality, 1999-2018

No. of NCDs	Model 1	Model 2	Model 3
S[0]	1.00 (reference)	1.00 (reference)	1.00 (reference)
S[1]	1.97 (1.62, 2.40)	1.51 (1.24, 1.84)	1.50 (1.23, 1.84)
S[2-4]	5.16 (4.36, 6.10)	2.43 (2.00, 2.95)	2.37 (1.96, 2.86)
S[5+]	16.03 (13.30, 19.33)	5.12 (4.10, 6.39)	4.49 (3.59, 5.60)

Values are presented as hazard ratio (95% confidence interval).

1 Model 1: mortality+number of NCDs; Model 2: model 1+age (20-39, 40-64, or ≥65 years), sex (male or female), race/ethnicity (Mexican American, other Hispanic, non-Hispanic White, non-Hispanic Black, or other race), and body mass index (kg/m^2^); Model 3: model 2+educational attainment (less than high school, high school, or more than high school), annual household income in dollars (<25,000, 25,000-75,000, or ≥75,000), physical activity (inactive, vigorous, or moderate), marital status (living together or single), smoking status (never, current, or former), and drinking status (never, current, or former).

**Table 5.** Stratified analyses^1^ of the associations between multimorbidity and all-cause mortality, 1999-2018

Variables^2^		HR_1_ (95% CI)	HR_2_ (95% CI)	HR_3_ (95% CI)	p-interaction
Sex					0.308
	Male (n=22,809)	1.50 (1.15, 1.95)	2.25 (1.73, 2.93)	3.86 (2.85, 5.24)	
	Female (n=24,542)	1.54 (1.10, 2.16)	2.56 (1.83, 3.57)	5.26 (3.71, 7.45)	
Age (yr)					<0.001
	20-39 (n=16,467)	1.73 (1.13, 2.65)	2.18 (1.27, 3.75)	17.85 (8.33, 38.28)	
	40-64 (n=19,497)	1.79 (1.22, 2.63)	3.13 (2.26, 4.34)	7.00 (4.85, 10.10)	
	≥65 (n=11,387)	0.86 (0.62, 1.20)	1.16 (0.90, 1.49)	2.10 (1.59, 2.78)	
Race/ethnicity					<0.001
	Mexican American (n=8,013)	1.01 (0.71, 1.45)	1.41 (0.98, 2.04)	2.18 (1.37, 3.47)	
	Other Hispanic (n=3,764)	2.73 (0.81, 9.18)	4.48 (1.68, 11.97)	12.28 (2.95, 51.01)	
	Non-Hispanic White (n=21,289)	1.58 (1.20, 2.08)	2.64 (2.03, 3.44)	4.84 (3.60, 6.49)	
	Non-Hispanic Black (n=9,898)	1.51 (0.98, 2.34)	1.92 (1.26, 2.93)	3.66 (2.23, 6.02)	
	Other race (n=4,387)	2.79 (0.78, 10.01)	2.72 (0.78, 9.52)	9.96 (2.33, 42.48)	
Educational attainment					0.010
	Less than high school (n=12,403)	1.99 (1.38, 2.87)	2.66 (1.93, 3.67)	4.71 (3.18, 6.96)	
	High school (n=10,998)	1.10 (0.76, 1.58)	2.01 (1.42, 2.84)	3.76 (2.58, 5.48)	
	More than high school (n=23,950)	1.55 (1.15, 2.08)	2.42 (1.79, 3.27)	4.77 (3.36, 6.77)	
Annual household income (US$)					0.703
	<25,000	1.69 (1.22, 2.36)	2.94 (2.10, 4.12)	5.33 (3.61, 7.86)	
	25,000-75,000	1.47 (1.09, 1.97)	2.25 (1.73, 2.93)	4.34 (3.26, 5.79)	
	≥75,000	1.40 (0.84, 2.35)	1.85 (1.16, 2.94)	3.70 (2.12, 6.44)	
Marital status					0.819
	Living together	1.44 (1.06, 1.97)	2.18 (1.63, 2.91)	4.10 (2.93, 5.72)	
	Single	1.58 (1.19, 2.09)	2.63 (2.01, 3.43)	4.98 (3.74, 6.63)	
Physical activity					0.662
	Never	1.30 (0.95, 1.77)	2.19 (1.63, 2.95)	4.14 (2.96, 5.80)	
	Vigorous	2.29 (1.07, 4.91)	3.80 (1.73, 8.36)	7.05 (3.16, 15.75)	
	Moderate	1.61 (1.20, 2.17)	2.29 (1.70, 3.10)	4.37 (3.09, 6.17)	
Smoking status					0.448
	Never	1.37 (1.01, 1.85)	2.09 (1.59, 2.74)	3.83 (2.79, 5.27)	
	Current	2.03 (1.48, 2.80)	3.25 (2.34, 4.51)	6.16 (4.15, 9.14)	
	Former	1.07 (0.74, 1.55)	1.69 (1.19, 2.40)	3.32 (2.28, 4.84)	
Drinking status					0.233
	Never	1.83 (1.15, 2.92)	2.29 (1.41, 3.74)	3.90 (2.35, 6.47)	
	Current	1.50 (1.20, 1.87)	2.38 (1.92, 2.95)	4.48 (3.45, 5.81)	
	Former	1.25 (0.72, 2.17)	2.33 (1.33, 4.05)	5.02 (2.76, 9.13)	

HR, hazard ratio; CI, confidence interval; NCD, non-communicable disease.

1 HRs compared with population with no diseases. HR1: S[1] (1 NCD), HR2: S[2-4] (2 to 4 NCDs), HR3: S[5+] (5 or more NCDs).

2 Adjusted for age (20-39, 40-64, or ≥65 years), sex (male or female), race/ethnicity (Mexican American, other Hispanic, non-Hispanic White, non-Hispanic Black, or other race), body mass index (kg/m^2^), educational attainment (<high school, high school, or >high school), annual household income in dollars (<25,000, 25,000-75,000, or ≥75,000), physical activity (inactive, vigorous, or moderate), marital status (living together or single), smoking status (never, current, or former), and drinking status (never, current, or former); Of note, variables examined in this table were not adjusted.

## SUPPLEMENTARY MATERIALS

Supplementary Material 1. Figure-Flowchart of participants in this study for cox regression.

Supplementary Material 2. eTable 1. Sample Size for Multimorbidity of NCDs among Adults in US by Sociodemographic, NHANES 1999-2000 (N(weighted %))

Supplementary Material 3. eTable 2. Sample Size for Multimorbidity of NCDs among Adults in US by Sociodemographic, NHANES 2001-2002 (N(weighted %))

Supplementary Material 4. eTable 3. Sample Size for Multimorbidity of NCDs among Adults in US by Sociodemographic, NHANES 2003-2004 (N(weighted %))

Supplementary Material 5. eTable 4. Sample Size for Multimorbidity of NCDs among Adults in US by Sociodemographic, NHANES 2005-2006 (N(weighted %))

Supplementary Material 6. eTable 5. Sample Size for Multimorbidity of NCDs among Adults in US by Sociodemographic, NHANES 2007-2008 (N(weighted %))

Supplementary Material 7. eTable 6. Sample Size for Multimorbidity of NCDs among Adults in US by Sociodemographic, NHANES 2009-2010 (N(weighted %))

Supplementary Material 8. eTable 7. Sample Size for Multimorbidity of NCDs among Adults in US by Sociodemographic, NHANES 2011-2012 (N(weighted %))

Supplementary Material 9. eTable 8. Sample Size for Multimorbidity of NCDs among Adults in US by Sociodemographic, NHANES 2013-2014 (N(weighted %))

Supplementary Material 10. eTable 9. Sample Size for Multimorbidity of NCDs among Adults in US by Sociodemographic, NHANES 2015-2016 (N(weighted %))

Supplementary Material 11. eTable 10. Crude Weighted Trends in every NCD among total Adults in US, NHANES 1999-2018 (N(weighted %))

Supplementary Material 12. eTable 11. Crude Weighted Trends in every cancer among total Adults in US, NHANES 1999-2018 (N(weighted %))

Supplementary Material 13. eTable 11 continued. Crude Weighted Trends in every cancer among total Adults in US, NHANES 1999-2018 (N(weighted %))

Supplementary Material 14. eTable 12. Weighted prevalence of every NCD in the age-subgroup and sex-subgroups, 2017-2018 (N(weighted %))
