# Supplementary material for: Trends and all-cause mortality associated with multimorbidity of non-communicable diseases among adults in the United States, 1999-2018: a retrospective cohort study
Source: Epidemiol Health. 2023 Feb 14;45:e2023023. doi: 10.4178/epih.e2023023 (PMC10586926; doi:10.4178/epih.e2023023)
Supplement: Supplementary Material 1. — Figure-Flowchart of participants in this study for cox regression. [file epih-45-e2023023-Supplementary-1.docx]

Supplementary Materials to:

**Trends and All-cause Mortality in Multimorbidity of Noncommunicable Diseases among Adults in the United States, 1999-2018: A Retrospective Cohort Study**


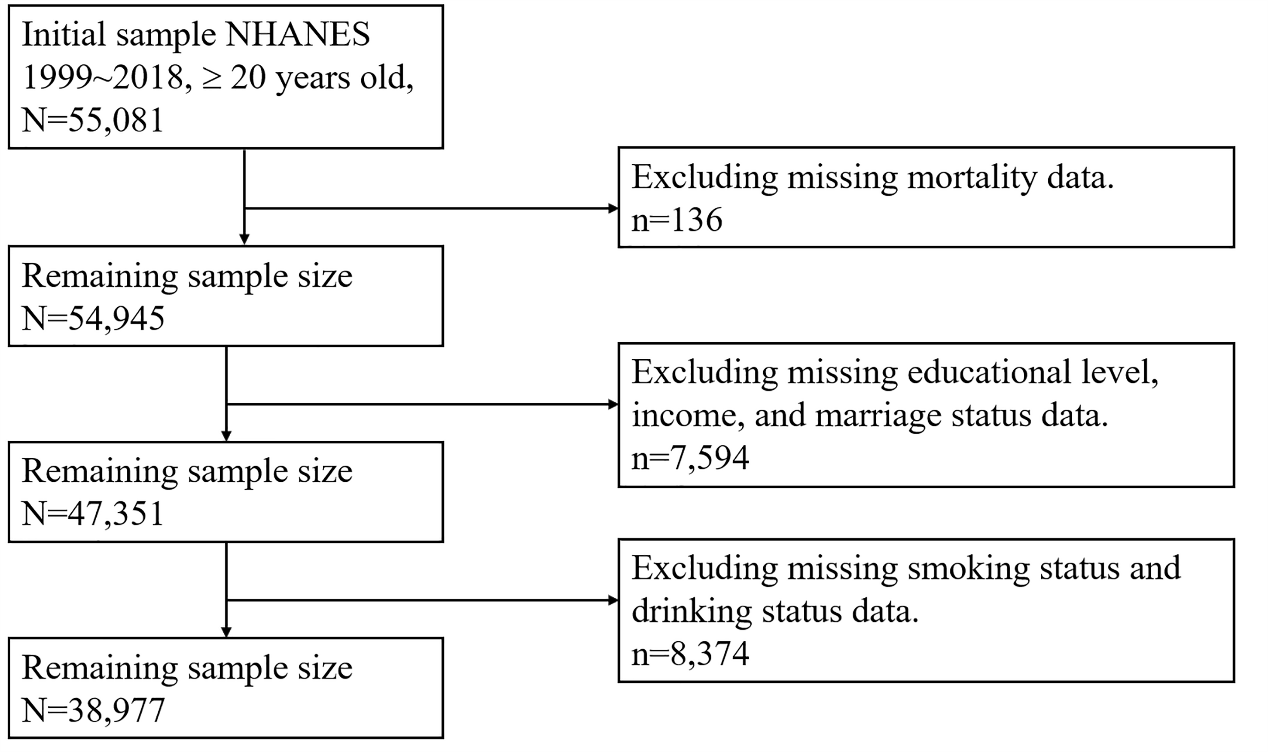


Supplementary Material 1. Figure-Flowchart of participants in this study for cox regression.
