# Supplementary material for: Trends and all-cause mortality associated with multimorbidity of non-communicable diseases among adults in the United States, 1999-2018: a retrospective cohort study
Source: Epidemiol Health. 2023 Feb 14;45:e2023023. doi: 10.4178/epih.e2023023 (PMC10586926; doi:10.4178/epih.e2023023)
Supplement: Supplementary Material 2. — eTable 1. Sample Size for Multimorbidity of NCDs among Adults in US by Sociodemographic, NHANES 1999-2000 (N(weighted %)) [file epih-45-e2023023-Supplementary-2.docx]

Supplementary Material 2: eTable 1. Sample Size for Multimorbidity of NCDs among Adults in US by Sociodemographic, NHANES 1999-2000 (N(weighted %))

|  |  |  | No. of Participants by Category of NCDs (Weighted %) | | | |
| --- | --- | --- | --- | --- | --- | --- |
|  | | Total | S[0] | S[1] | S[2~4] | s[5+] |
| Overall | | 4880(100.0) | 1360(33.8) | 1247(26.7) | 1910(33.8) | 363(5.7) |
| Age | |  |  |  |  |  |
|  | 20~39 | 1695(44.1) | 887(68.8) | 542(51.5) | 256(20.1) | 10(6.2) |
|  | 40~64 | 1793(39.7) | 367(28.1) | 463(38.9) | 830(50.8) | 133(46.7) |
|  | 65~ | 1392(16.1) | 106(3.0) | 242(9.7) | 824(29.1) | 220(47.1) |
| Sex | |  |  |  |  |  |
|  | Male | 2269(47.7) | 682(52.0) | 583(47.9) | 831(43.8) | 173(45.5) |
|  | Female | 2611(52.3) | 678(48.0) | 664(52.1) | 1079(56.2) | 190(54.5) |
| Race /ethnicity | |  |  |  |  |  |
|  | Mexican American | 1282(6.4) | 415(8.4) | 344(6.7) | 456(4.8) | 67(2.2) |
|  | Other Hispanic | 310(8.1) | 98(9.2) | 85(9.0) | 107(6.4) | 20(7.1) |
|  | Non-Hispanic White | 2214(70.1) | 569(67.4) | 549(68.7) | 894(72.3) | 202(79.3) |
|  | Non-Hispanic Black | 910(10.9) | 226(9.9) | 230(11.4) | 388(11.7) | 66(8.9) |
|  | Other Race | 164(4.6) | 52(5.1) | 39(4.2) | 65(4.8) | 8(2.5) |
| Annual household income, $ | |  |  |  |  |  |
|  | <25000 | 1559(29.2) | 363(24.6) | 349(25.3) | 677(33.8) | 170(48.2) |
|  | 25000~75000 | 1865(48.9) | 555(49.6) | 502(50.7) | 697(47.6) | 111(44.1) |
|  | ≥75000 | 657(21.9) | 233(25.8) | 191(24) | 212(18.6) | 21(7.7) |
| Educational attainment | |  |  |  |  |  |
|  | <High School | 1896(24.2) | 473(21.9) | 454(21.2) | 800(26.8) | 169(36.6) |
|  | High School | 1097(25.9) | 300(23.1) | 250(22.7) | 449(30.1) | 98(33.0) |
|  | >High School | 49.9(1863) | 578(55.1) | 541(56.2) | 650(43.1) | 94(30.4) |
| Marriage Status | |  |  |  |  |  |
|  | Live together | 2635(62.3) | 727(59.9) | 682(60.4) | 1020(64.8) | 206(69.3) |
|  | Single | 1700(37.7) | 470(40.1) | 424(39.6) | 692(35.2) | 114(30.7) |
| Physical activity | |  |  |  |  |  |
|  | Never | 2578(42.2) | 599(33.9) | 647(41.8) | 1085(47.6) | 247(61.7) |
|  | Vigorous | 565(15.3) | 220(19.3) | 157(16.5) | 165(11.5) | 23(8.2) |
|  | Moderate | 1737(42.5) | 541(46.8) | 443(41.7) | 660(40.9) | 93(30.1) |
| Smoking status | |  |  |  |  |  |
|  | Never | 2566(50.5) | 780(54.3) | 674(52.2) | 947(46.4) | 165(44.7) |
|  | Current | 999(25.2) | 362(31.2) | 260(24.4) | 325(21.1) | 52(18.2) |
|  | Former | 1300(24.3) | 214(14.5) | 308(23.4) | 632(32.5) | 146(37.1) |
| Drinking status | |  |  |  |  |  |
|  | Never | 642(13.3) | 156(10.9) | 154(11.9) | 271(15.1) | 61(24.9) |
|  | Current | 2656(78.2) | 827(84.3) | 731(81.3) | 970(72.6) | 128(58.3) |
|  | Former | 407(8.5) | 69(4.8) | 85(6.8) | 204(12.3) | 49(16.8) |
