# Supplementary material for: Trends and all-cause mortality associated with multimorbidity of non-communicable diseases among adults in the United States, 1999-2018: a retrospective cohort study
Source: Epidemiol Health. 2023 Feb 14;45:e2023023. doi: 10.4178/epih.e2023023 (PMC10586926; doi:10.4178/epih.e2023023)
Supplement: Supplementary Material 3. — eTable 2. Sample Size for Multimorbidity of NCDs among Adults in US by Sociodemographic, NHANES 2001-2002 (N(weighted %)) [file epih-45-e2023023-Supplementary-3.docx]

Supplementary Material 3: eTable 2. Sample Size for Multimorbidity of NCDs among Adults in US by Sociodemographic, NHANES 2001-2002 (N(weighted %))

|  |  |  | No. of Participants by Category of NCDs (Weighted %) | | | |
| --- | --- | --- | --- | --- | --- | --- |
|  | | Total | S[0] | S[1] | S[2~4] | s[5+] |
| Overall | | 5411(100.0) | 1525(31.5) | 1315(26.6) | 2051(34.4) | 520(7.6) |
| Age | |  |  |  |  |  |
|  | 20~39 | 1925(41.1) | 1018(66.4) | 562(45.8) | 331(22.2) | 14(5.9) |
|  | 40~64 | 2023(43.5) | 404(30.6) | 547(46.8) | 886(52.1) | 186(46.0) |
|  | 65~ | 1463(15.4) | 103(3.1) | 206(7.4) | 834(25.7) | 320(48.1) |
| Sex | |  |  |  |  |  |
|  | Male | 2536(47.8) | 772(51.6) | 612(49.0) | 929(45.5) | 223(38.1) |
|  | Female | 2875(52.2) | 753(48.4) | 703(51.0) | 1122(54.5) | 297(61.9) |
| Race /ethnicity | |  |  |  |  |  |
|  | Mexican American | 1113(7.2) | 430(11.4) | 284(6.9) | 342(4.5) | 57(2.2) |
|  | Other Hispanic | 237(6.0) | 72(6.0) | 71(7.2) | 81(5.5) | 13(4.5) |
|  | Non-Hispanic White | 2858(71.3) | 694(66.1) | 657(70.3) | 1160(74.6) | 347(81.1) |
|  | Non-Hispanic Black | 1012(10.9) | 257(10.3) | 253(11.3) | 407(11.2) | 95(10.7) |
|  | Other Race | 191(4.6) | 72(6.1) | 50(4.4) | 61(4.2) | 8(1.4) |
| Annual household income, $ | |  |  |  |  |  |
|  | <25000 | 1582(25.4) | 347(21.3) | 359(22.6) | 672(28.3) | 204(39.9) |
|  | 25000~75000 | 2212(45.8) | 676(46.0) | 551(48.0) | 789(44.6) | 196(43.2) |
|  | ≥75000 | 1062(28.7) | 354(32.7) | 271(29.4) | 368(27.1) | 69(16.9) |
| Educational attainment | |  |  |  |  |  |
|  | <High School | 1667(19.6) | 447(18.5) | 366(16.7) | 652(20.3) | 202(31.7) |
|  | High School | 1265(25.3) | 344(24.5) | 311(24.7) | 487(26.3) | 123(27.0) |
|  | >High School | 2458(55) | 728(56.9) | 634(58.7) | 904(53.5) | 192(41.3) |
| Marriage Status | |  |  |  |  |  |
|  | Live together | 3346(64.0) | 966(63.3) | 825(63.0) | 1282(67.4) | 273(55.7) |
|  | Single | 2057(36.0) | 558(36.7) | 486(37.0) | 768(32.6) | 245(44.3) |
| Physical activity | |  |  |  |  |  |
|  | Never | 2467(36.4) | 576(28.6) | 571(35.0) | 1001(40.1) | 319(57.4) |
|  | Vigorous | 653(13.3) | 241(16.7) | 160(12.9) | 218(11.9) | 34(6.6) |
|  | Moderate | 2291(50.3) | 708(54.7) | 584(52.1) | 832(48.0) | 167(36.0) |
| Smoking status | |  |  |  |  |  |
|  | Never | 2801(50.9) | 899(57.2) | 666(49.5) | 1027(48.6) | 209(39.8) |
|  | Current | 1171(24.6) | 374(26.4) | 338(26.9) | 385(22.8) | 74(17.0) |
|  | Former | 1424(24.6) | 251(16.4) | 304(23.7) | 633(28.6) | 236(43.3) |
| Drinking status | |  |  |  |  |  |
|  | Never | 721(15.0) | 192(14.2) | 169(14.1) | 294(16.1) | 66(17.9) |
|  | Current | 2981(76.9) | 923(81.7) | 776(78.3) | 1083(74.1) | 199(62.8) |
|  | Former | 428(8.1) | 68(4.2) | 97(7.5) | 192(9.8) | 71(19.3) |
