# Supplementary material for: Trends and all-cause mortality associated with multimorbidity of non-communicable diseases among adults in the United States, 1999-2018: a retrospective cohort study
Source: Epidemiol Health. 2023 Feb 14;45:e2023023. doi: 10.4178/epih.e2023023 (PMC10586926; doi:10.4178/epih.e2023023)
Supplement: Supplementary Material 4. — eTable 3. Sample Size for Multimorbidity of NCDs among Adults in US by Sociodemographic, NHANES 2003-2004 (N(weighted %)) [file epih-45-e2023023-Supplementary-4.docx]

Supplementary Material 4: eTable 3. Sample Size for Multimorbidity of NCDs among Adults in US by Sociodemographic, NHANES 2003-2004 (N(weighted %))

|  |  |  | No. of Participants by Category of NCDs (Weighted %) | | | |
| --- | --- | --- | --- | --- | --- | --- |
|  | | Total | S[0] | S[1] | S[2~4] | s[5+] |
| Overall | | 5041(100.0) | 1186(26.8) | 1181(25.5) | 2038(37.6) | 636(10.1) |
| Age | |  |  |  |  |  |
|  | 20~39 | 1742(38.8) | 792(65.8) | 582(50.8) | 351(20.9) | 17(4.1) |
|  | 40~64 | 1805(44.1) | 309(31.4) | 405(41.0) | 897(55.5) | 194(43.1) |
|  | 65~ | 1494(17.1) | 85(2.8) | 194(8.2) | 790(23.6) | 425(52.8) |
| Sex | |  |  |  |  |  |
|  | Male | 2418(47.9) | 633(52.5) | 571(48.5) | 945(46.0) | 269(41.6) |
|  | Female | 2623(52.1) | 553(47.5) | 610(51.5) | 1093(54.0) | 367(58.4) |
| Race /ethnicity | |  |  |  |  |  |
|  | Mexican American | 985(7.8) | 253(10.7) | 256(9.1) | 403(6.1) | 73(2.8) |
|  | Other Hispanic | 152(3.6) | 51(5.3) | 51(4.5) | 40(2.5) | 10(1.3) |
|  | Non-Hispanic White | 2689(71.8) | 570(65.2) | 572(69.4) | 1108(75.1) | 439(82.8) |
|  | Non-Hispanic Black | 994(11.2) | 226(9.9) | 259(12.4) | 415(12.1) | 94(8.6) |
|  | Other Race | 221(5.6) | 86(8.9) | 43(4.6) | 72(4.1) | 20(4.5) |
| Annual household income, $ | |  |  |  |  |  |
|  | <25000 | 1637(24.2) | 317(20.3) | 358(22.7) | 673(23.9) | 289(40.2) |
|  | 25000~75000 | 2151(49.3) | 517(47.5) | 518(51.1) | 877(50.0) | 239(47.3) |
|  | ≥75000 | 907(26.4) | 265(32.3) | 219(26.2) | 369(26.1) | 54(12.6) |
| Educational attainment | |  |  |  |  |  |
|  | <High School | 1487(18.3) | 276(15.3) | 343(17.6) | 633(18.3) | 235(28.5) |
|  | High School | 1269(26.9) | 300(24.8) | 284(26.1) | 525(29.2) | 160(26.1) |
|  | >High School | 2271(54.7) | 609(60.0) | 549(56.3) | 873(52.5) | 240(45.4) |
| Marriage Status | |  |  |  |  |  |
|  | Live together | 2993(63.3) | 687(59.8) | 712(62.6) | 1257(67.0) | 337(59.9) |
|  | Single | 2045(36.7) | 498(40.2) | 468(37.4) | 780(33.0) | 299(40.1) |
| Physical activity | |  |  |  |  |  |
|  | Never | 2166(35.5) | 405(27.5) | 473(33.6) | 928(38.0) | 360(51.7) |
|  | Vigorous | 350(7.5) | 132(11.0) | 96(8.5) | 104(5.5) | 18(2.9) |
|  | Moderate | 2525(57.1) | 649(61.5) | 612(57.9) | 1006(56.5) | 258(45.4) |
| Smoking status | |  |  |  |  |  |
|  | Never | 2538(49.5) | 675(55.7) | 625(51.4) | 988(47.2) | 250(37.2) |
|  | Current | 1131(25.5) | 318(29.1) | 307(29.4) | 409(22.4) | 97(17.8) |
|  | Former | 1365(24.9) | 190(15.3) | 247(19.2) | 639(30.4) | 289(45.0) |
| Drinking status | |  |  |  |  |  |
|  | Never | 631(13.1) | 127(11.9) | 129(10.2) | 286(15.1) | 89(17.0) |
|  | Current | 2699(76.9) | 701(82.7) | 700(81.1) | 1064(73.9) | 234(59.5) |
|  | Former | 429(10.0) | 58(5.4) | 92(8.6) | 184(11.0) | 95(23.5) |
