# Supplementary material for: Trends and all-cause mortality associated with multimorbidity of non-communicable diseases among adults in the United States, 1999-2018: a retrospective cohort study
Source: Epidemiol Health. 2023 Feb 14;45:e2023023. doi: 10.4178/epih.e2023023 (PMC10586926; doi:10.4178/epih.e2023023)
Supplement: Supplementary Material 5. — eTable 4. Sample Size for Multimorbidity of NCDs among Adults in US by Sociodemographic, NHANES 2005-2006 (N(weighted %)) [file epih-45-e2023023-Supplementary-5.docx]

Supplementary Material 5: eTable 4. Sample Size for Multimorbidity of NCDs among Adults in US by Sociodemographic, NHANES 2005-2006 (N(weighted %))

|  |  |  | No. of Participants by Category of NCDs (Weighted %) | | | |
| --- | --- | --- | --- | --- | --- | --- |
|  | | Total | S[0] | S[1] | S[2~4] | s[5+] |
| Overall | | 4979(100.0) | 1250(24.9) | 1192(24.7) | 1937(39.3) | 600(11.1) |
| Age | |  |  |  |  |  |
|  | 20~39 | 1923(38.0) | 874(66.4) | 609(49.1) | 424(22.8) | 16(3.4) |
|  | 40~64 | 1867(44.8) | 316(30.7) | 432(43.5) | 881(53.8) | 238(47.9) |
|  | 65~ | 1189(17.2) | 60(2.8) | 151(7.4) | 632(23.4) | 346(48.7) |
| Sex | |  |  |  |  |  |
|  | Male | 2387(48.1) | 615(52.3) | 600(51.5) | 926(46.4) | 246(37.0) |
|  | Female | 2592(51.9) | 635(47.7) | 592(48.5) | 1011(53.6) | 354(63.0) |
| Race /ethnicity | |  |  |  |  |  |
|  | Mexican American | 1003(8.0) | 383(13.6) | 273(8.9) | 301(5.4) | 46(2.2) |
|  | Other Hispanic | 154(3.4) | 48(4.0) | 43(4.0) | 58(3.3) | 5(0.9) |
|  | Non-Hispanic White | 2495(71.8) | 514(64.4) | 549(69.9) | 1047(75.2) | 385(80.8) |
|  | Non-Hispanic Black | 1123(11.5) | 238(10.9) | 277(12) | 464(11.7) | 144(10.8) |
|  | Other Race | 204(5.4) | 67(7.1) | 50(5.2) | 67(4.4) | 20(5.2) |
| Annual household income, $ | |  |  |  |  |  |
|  | <25000 | 1388(20.5) | 314(17.8) | 278(16.8) | 564(21.2) | 232(31.7) |
|  | 25000~75000 | 2316(48.6) | 597(50.7) | 559(46.9) | 894(48.6) | 266(48.0) |
|  | ≥75000 | 1105(30.9) | 287(31.5) | 311(36.3) | 426(30.2) | 81(20.3) |
| Educational attainment | |  |  |  |  |  |
|  | <High School | 1394(17.7) | 375(18.1) | 304(16.2) | 527(17.2) | 188(22.1) |
|  | High School | 1181(24.9) | 275(23.4) | 256(22.0) | 486(26.5) | 164(29.3) |
|  | >High School | 2395(57.4) | 599(58.6) | 631(61.8) | 918(56.3) | 247(48.6) |
| Marriage Status | |  |  |  |  |  |
|  | Live together | 3095(65.5) | 804(63.4) | 769(68.4) | 1199(66.5) | 323(60.1) |
|  | Single | 1877(34.5) | 445(36.6) | 422(31.6) | 734(33.5) | 276(39.9) |
| Physical activity | |  |  |  |  |  |
|  | Never | 1976(33.0) | 439(27.0) | 423(29.2) | 783(34.6) | 331(49.2) |
|  | Vigorous | 357(7.4) | 139(11.0) | 97(9.0) | 99(5.3) | 22(3.7) |
|  | Moderate | 2646(59.5) | 672(62.0) | 672(61.8) | 1055(60.1) | 247(47.1) |
| Smoking status | |  |  |  |  |  |
|  | Never | 2625(51.0) | 757(58.0) | 648(51.5) | 973(48.9) | 247(41.7) |
|  | Current | 1089(24.0) | 298(26.6) | 276(25.9) | 411(22.7) | 104(19.0) |
|  | Former | 1259(24.9) | 194(15.4) | 266(22.7) | 550(28.4) | 249(39.3) |
| Drinking status | |  |  |  |  |  |
|  | Never | 604(11.9) | 165(13.0) | 137(10.6) | 212(10.5) | 90(17.7) |
|  | Current | 2822(79.1) | 753(81.6) | 732(82.5) | 1082(79.3) | 255(63.9) |
|  | Former | 443(9.0) | 70(5.3) | 77(6.9) | 201(10.2) | 95(18.4) |
