# Supplementary material for: Trends and all-cause mortality associated with multimorbidity of non-communicable diseases among adults in the United States, 1999-2018: a retrospective cohort study
Source: Epidemiol Health. 2023 Feb 14;45:e2023023. doi: 10.4178/epih.e2023023 (PMC10586926; doi:10.4178/epih.e2023023)
Supplement: Supplementary Material 6. — eTable 5. Sample Size for Multimorbidity of NCDs among Adults in US by Sociodemographic, NHANES 2007-2008 (N(weighted %)) [file epih-45-e2023023-Supplementary-6.docx]

Supplementary Material 6: eTable 5. Sample Size for Multimorbidity of NCDs among Adults in US by Sociodemographic, NHANES 2007-2008 (N(weighted %))

|  |  |  | No. of Participants by Category of NCDs (Weighted %) | | | |
| --- | --- | --- | --- | --- | --- | --- |
|  | | Total | S[0] | S[1] | S[2~4] | s[5+] |
| Overall | | 5935(100.0) | 1254(24.1) | 1253(23.1) | 2410(39.9) | 1018(12.9) |
| Age | |  |  |  |  |  |
|  | 20~39 | 1910(37.5) | 826(67.6) | 586(51.2) | 449(21.7) | 49(6.0) |
|  | 40~64 | 2469(45.7) | 371(29.9) | 517(42.7) | 1174(57.0) | 407(45.3) |
|  | 65~ | 1556(16.8) | 57(2.5) | 150(6.2) | 787(21.3) | 562(48.7) |
| Sex | |  |  |  |  |  |
|  | Male | 2910(48.2) | 674(53.1) | 630(48.4) | 1147(46.5) | 459(43.7) |
|  | Female | 3025(51.8) | 580(46.9) | 623(51.6) | 1263(53.5) | 559(56.3) |
| Race /ethnicity | |  |  |  |  |  |
|  | Mexican American | 1033(8.4) | 297(12.4) | 279(10.8) | 342(5.9) | 115(4.0) |
|  | Other Hispanic | 666(4.9) | 148(5.6) | 178(6.1) | 248(4.1) | 92(3.5) |
|  | Non-Hispanic White | 2761(69.4) | 485(62.9) | 505(64.9) | 1215(73.8) | 556(75.9) |
|  | Non-Hispanic Black | 1227(11.3) | 251(11.3) | 230(11) | 518(11.1) | 228(12.2) |
|  | Other Race | 248(6.1) | 73(7.8) | 61(7.1) | 87(5) | 27(4.4) |
| Annual household income, $ | |  |  |  |  |  |
|  | <25000 | 1846(22.6) | 345(21.1) | 356(20.0) | 711(20.7) | 434(35.7) |
|  | 25000~75000 | 2611(45.3) | 563(44.4) | 559(45.4) | 1104(47.2) | 385(41.3) |
|  | ≥75000 | 1232(32.1) | 294(34.5) | 286(34.6) | 509(32.1) | 143(23.0) |
| Educational attainment | |  |  |  |  |  |
|  | <High School | 1861(20.5) | 354(18.9) | 380(19.8) | 739(19.4) | 388(28.4) |
|  | High School | 1462(25.4) | 321(25.5) | 296(24.0) | 593(25.7) | 252(26.9) |
|  | >High School | 2605(54) | 577(55.6) | 576(56.2) | 1075(54.8) | 377(44.7) |
| Marriage Status | |  |  |  |  |  |
|  | Live together | 3517(63.4) | 738(60.5) | 750(61.7) | 1474(66.6) | 555(61.9) |
|  | Single | 2414(36.6) | 514(39.5) | 502(38.3) | 935(33.4) | 463(38.1) |
| Physical activity | |  |  |  |  |  |
|  | Never | 3498(53.4) | 647(47.4) | 694(51.8) | 1436(53.9) | 721(65.7) |
|  | Vigorous | 233(4.4) | 65(5.2) | 58(5.0) | 87(3.9) | 23(3.0) |
|  | Moderate | 2204(42.2) | 542(47.4) | 501(43.2) | 887(42.1) | 274(31.3) |
| Smoking status | |  |  |  |  |  |
|  | Never | 3127(53.2) | 747(57.9) | 716(57.1) | 1231(51.3) | 433(43.0) |
|  | Current | 1319(22.8) | 343(26.6) | 310(25.8) | 477(20.3) | 189(18.3) |
|  | Former | 1482(24) | 164(15.5) | 226(17.1) | 698(28.4) | 394(38.7) |
| Drinking status | |  |  |  |  |  |
|  | Never | 789(13.6) | 134(10.9) | 141(11.1) | 355(14.8) | 159(20.0) |
|  | Current | 3323(78.5) | 803(84.7) | 802(85.2) | 1303(75.4) | 415(62.8) |
|  | Former | 423(7.9) | 48(4.3) | 49(3.7) | 202(9.8) | 124(17.2) |
