# Supplementary material for: Trends and all-cause mortality associated with multimorbidity of non-communicable diseases among adults in the United States, 1999-2018: a retrospective cohort study
Source: Epidemiol Health. 2023 Feb 14;45:e2023023. doi: 10.4178/epih.e2023023 (PMC10586926; doi:10.4178/epih.e2023023)
Supplement: Supplementary Material 7. — eTable 6. Sample Size for Multimorbidity of NCDs among Adults in US by Sociodemographic, NHANES 2009-2010 (N(weighted %)) [file epih-45-e2023023-Supplementary-7.docx]

Supplementary Material 7: eTable 6. Sample Size for Multimorbidity of NCDs among Adults in US by Sociodemographic, NHANES 2009-2010 (N(weighted %))

|  |  |  | No. of Participants by Category of NCDs (Weighted %) | | | |
| --- | --- | --- | --- | --- | --- | --- |
|  | | Total | S[0] | S[1] | S[2~4] | s[5+] |
| Overall | | 6218(100.0) | 1377(25.1) | 1365(23.3) | 2487(38.8) | 989(12.9) |
| Age | |  |  |  |  |  |
|  | 20~39 | 2083(37.0) | 889(64.8) | 632(48.2) | 535(23.6) | 27(3.3) |
|  | 40~64 | 2612(45.3) | 439(33.2) | 577(44.5) | 1192(52.9) | 404(47.1) |
|  | 65~ | 1523(17.7) | 49(1.9) | 156(7.3) | 760(23.5) | 558(49.6) |
| Sex | |  |  |  |  |  |
|  | Male | 3006(48.2) | 703(50.6) | 677(50.5) | 1186(47.0) | 440(43.1) |
|  | Female | 3212(51.8) | 674(49.4) | 688(49.5) | 1301(53.0) | 549(56.9) |
| Race /ethnicity | |  |  |  |  |  |
|  | Mexican American | 1140(8.6) | 314(11.7) | 289(10.3) | 421(7.0) | 116(4.2) |
|  | Other Hispanic | 632(5.0) | 168(7.0) | 130(4.5) | 245(4.6) | 89(3.4) |
|  | Non-Hispanic White | 2976(67.9) | 583(61.1) | 614(65.5) | 1232(71.2) | 547(75.5) |
|  | Non-Hispanic Black | 1122(11.4) | 189(9.0) | 258(12.5) | 478(11.8) | 197(12.8) |
|  | Other Race | 348(7.1) | 123(11.2) | 74(7.2) | 111(5.4) | 40(4.1) |
| Annual household income, $ | |  |  |  |  |  |
|  | <25000 | 1803(20.8) | 347(19.0) | 356(18.1) | 748(21.3) | 352(27.5) |
|  | 25000~75000 | 2715(44.9) | 570(42.4) | 628(46.1) | 1069(44.7) | 448(48.3) |
|  | ≥75000 | 1372(34.3) | 359(38.6) | 312(35.8) | 558(34.0) | 143(24.2) |
| Educational attainment | |  |  |  |  |  |
|  | <High School | 1776(19.1) | 366(19.1) | 361(16.7) | 715(18.1) | 334(26.0) |
|  | High School | 1426(22.9) | 296(19.8) | 314(22.5) | 582(24.6) | 234(24.3) |
|  | >High School | 3001(58.1) | 709(61.1) | 689(60.7) | 1186(57.3) | 417(49.6) |
| Marriage Status | |  |  |  |  |  |
|  | Live together | 3669(63.3) | 809(60.2) | 810(62.6) | 1491(65.7) | 559(63.0) |
|  | Single | 2545(36.7) | 567(39.8) | 554(37.4) | 995(34.3) | 429(37.0) |
| Physical activity | |  |  |  |  |  |
|  | Never | 3835(58.4) | 781(56.6) | 799(56.9) | 1560(58.0) | 695(65.8) |
|  | Vigorous | 259(4.2) | 69(4.7) | 73(4.7) | 95(4.0) | 22(3.1) |
|  | Moderate | 2124(37.4) | 527(38.7) | 493(38.5) | 832(38.0) | 272(31.0) |
| Smoking status | |  |  |  |  |  |
|  | Never | 3352(55.4) | 838(60.6) | 796(59.0) | 1299(54.0) | 419(42.9) |
|  | Current | 1346(20.3) | 336(23.2) | 337(23.0) | 497(17.9) | 176(17.3) |
|  | Former | 1520(24.3) | 203(16.2) | 232(18.0) | 691(28.1) | 394(39.8) |
| Drinking status | |  |  |  |  |  |
|  | Never | 677(11.4) | 133(10.8) | 126(9.1) | 283(11.9) | 135(15.3) |
|  | Current | 3666(82.3) | 877(84.4) | 879(86.3) | 1438(81.8) | 472(72.0) |
|  | Former | 361(6.3) | 51(4.8) | 60(4.7) | 150(6.2) | 100(12.7) |
