# Supplementary material for: Trends and all-cause mortality associated with multimorbidity of non-communicable diseases among adults in the United States, 1999-2018: a retrospective cohort study
Source: Epidemiol Health. 2023 Feb 14;45:e2023023. doi: 10.4178/epih.e2023023 (PMC10586926; doi:10.4178/epih.e2023023)
Supplement: Supplementary Material 8. — eTable 7. Sample Size for Multimorbidity of NCDs among Adults in US by Sociodemographic, NHANES 2011-2012 (N(weighted %)) [file epih-45-e2023023-Supplementary-8.docx]

Supplementary Material 8: eTable 7. Sample Size for Multimorbidity of NCDs among Adults in US by Sociodemographic, NHANES 2011-2012 (N(weighted %))

|  |  |  | No. of Participants by Category of NCDs (Weighted %) | | | |
| --- | --- | --- | --- | --- | --- | --- |
|  | | Total | S[0] | S[1] | S[2~4] | s[5+] |
| Overall | | 5560(100.0) | 1238(22.7) | 1234(23.9) | 2251(40.4) | 837(12.9) |
| Age | |  |  |  |  |  |
|  | 20~39 | 1957(36.3) | 821(66.4) | 626(50.1) | 476(21.4) | 34(4.0) |
|  | 40~64 | 2353(46.0) | 371(30.8) | 487(43.3) | 1129(55.4) | 366(48.5) |
|  | 65~ | 1250(17.7) | 46(2.7) | 121(6.6) | 646(23.1) | 437(47.5) |
| Sex | |  |  |  |  |  |
|  | Male | 2740(48.0) | 638(49.4) | 627(49.8) | 1113(48.2) | 362(41.2) |
|  | Female | 2820(52.0) | 600(50.6) | 607(50.2) | 1138(51.8) | 475(58.8) |
| Race /ethnicity | |  |  |  |  |  |
|  | Mexican American | 540(7.7) | 146(10.5) | 129(8.7) | 208(6.6) | 57(4.0) |
|  | Other Hispanic | 578(6.6) | 111(7.8) | 129(7.2) | 255(6.2) | 83(4.8) |
|  | Non-Hispanic White | 2041(66.5) | 364(58.9) | 438(65.6) | 856(69.1) | 383(73.5) |
|  | Non-Hispanic Black | 1455(11.5) | 268(10.9) | 311(11.4) | 620(11.5) | 256(12.7) |
|  | Other Race | 946(7.7) | 349(11.9) | 227(7.1) | 312(6.6) | 58(5.1) |
| Annual household income, $ | |  |  |  |  |  |
|  | <25000 | 1752(22.8) | 315(22.7) | 359(19.4) | 708(21.6) | 370(33.3) |
|  | 25000~75000 | 2185(42.7) | 479(38.8) | 483(42.7) | 905(44.1) | 318(44.8) |
|  | ≥75000 | 1331(34.5) | 362(38.4) | 329(37.8) | 531(34.3) | 109(21.9) |
| Educational attainment | |  |  |  |  |  |
|  | <High School | 1332(16.7) | 223(14.6) | 250(12.9) | 585(17.7) | 274(24.1) |
|  | High School | 1169(20.1) | 220(15.7) | 267(21.8) | 492(20.8) | 190(22.4) |
|  | >High School | 3054(63.2) | 795(69.7) | 715(65.3) | 1171(61.5) | 373(53.5) |
| Marriage Status | |  |  |  |  |  |
|  | Live together | 3123(61.4) | 654(56.1) | 739(64.9) | 1325(63.7) | 405(57.3) |
|  | Single | 2430(38.6) | 582(43.9) | 493(35.1) | 925(36.3) | 430(42.7) |
| Physical activity | |  |  |  |  |  |
|  | Never | 3616(60.9) | 796(58.8) | 777(60.7) | 1444(60.6) | 599(65.9) |
|  | Vigorous | 227(4.1) | 52(5.0) | 59(4.2) | 95(4.0) | 21(2.4) |
|  | Moderate | 1717(35.0) | 390(36.1) | 398(35.1) | 712(35.4) | 217(31.7) |
| Smoking status | |  |  |  |  |  |
|  | Never | 3184(56.3) | 853(68.0) | 743(61.1) | 1205(50.7) | 383(44.5) |
|  | Current | 1108(19.8) | 236(19.3) | 276(18.3) | 439(21.4) | 157(18.6) |
|  | Former | 1259(23.9) | 144(12.7) | 214(20.6) | 604(27.9) | 297(36.8) |
| Drinking status | |  |  |  |  |  |
|  | Never | 696(11.8) | 139(10.8) | 139(10.9) | 302(11.8) | 116(15.7) |
|  | Current | 3149(83.3) | 753(86.1) | 748(85.7) | 1257(82.9) | 391(75.1) |
|  | Former | 266(4.8) | 34(3.1) | 47(3.4) | 114(5.4) | 71(9.2) |
