# Supplementary material for: Trends and all-cause mortality associated with multimorbidity of non-communicable diseases among adults in the United States, 1999-2018: a retrospective cohort study
Source: Epidemiol Health. 2023 Feb 14;45:e2023023. doi: 10.4178/epih.e2023023 (PMC10586926; doi:10.4178/epih.e2023023)
Supplement: Supplementary Material 9. — eTable 8. Sample Size for Multimorbidity of NCDs among Adults in US by Sociodemographic, NHANES 2013-2014 (N(weighted %)) [file epih-45-e2023023-Supplementary-9.docx]

Supplementary Material 9: eTable 8. Sample Size for Multimorbidity of NCDs among Adults in US by Sociodemographic, NHANES 2013-2014 (N(weighted %))

|  |  |  | No. of Participants by Category of NCDs (Weighted %) | | | |
| --- | --- | --- | --- | --- | --- | --- |
|  | | Total | S[0] | S[1] | S[2~4] | s[5+] |
| Overall | | 5769(100.0) | 1190(21.5) | 1233(21.1) | 2329(40.9) | 1017(16.5) |
| Age | |  |  |  |  |  |
|  | 20~39 | 1954(36.2) | 765(64.6) | 594(51.1) | 553(26.5) | 42(4.2) |
|  | 40~64 | 2509(45.1) | 396(33.6) | 519(42.6) | 1158(51.8) | 436(46.9) |
|  | 65~ | 1306(18.6) | 29(1.8) | 120(6.2) | 618(21.7) | 539(48.9) |
| Sex | |  |  |  |  |  |
|  | Male | 2758(48.1) | 615(50.8) | 637(53.0) | 1106(48.2) | 400(37.8) |
|  | Female | 3011(51.9) | 575(49.2) | 596(47.0) | 1223(51.8) | 617(62.2) |
| Race /ethnicity | |  |  |  |  |  |
|  | Mexican American | 767(9.2) | 166(11.9) | 193(12.2) | 305(7.8) | 103(5.0) |
|  | Other Hispanic | 508(5.5) | 116(7.1) | 127(7.0) | 195(5.0) | 70(2.9) |
|  | Non-Hispanic White | 2472(65.8) | 429(57.8) | 443(58.9) | 1032(68.6) | 568(78.1) |
|  | Non-Hispanic Black | 1177(11.4) | 207(10.8) | 252(12.4) | 510(11.9) | 208(9.9) |
|  | Other Race | 845(8.1) | 272(12.4) | 218(9.5) | 287(6.7) | 68(4.1) |
| Annual household income, $ | |  |  |  |  |  |
|  | <25000 | 1607(21.6) | 294(20.9) | 277(18.0) | 632(20.2) | 404(30.9) |
|  | 25000~75000 | 2353(42.0) | 464(37.8) | 511(40.1) | 969(43.6) | 409(45.6) |
|  | ≥75000 | 1533(36.4) | 363(41.3) | 383(41.9) | 618(36.2) | 169(23.5) |
| Educational attainment | |  |  |  |  |  |
|  | <High School | 1246(15.3) | 236(15.3) | 265(15.6) | 486(14.3) | 259(17.6) |
|  | High School | 1303(21.9) | 262(21.8) | 236(17.8) | 541(22.8) | 264(25.0) |
|  | >High School | 3213(62.8) | 690(62.9) | 732(66.6) | 1299(63.0) | 492(57.5) |
| Marriage Status | |  |  |  |  |  |
|  | Live together | 3382(62.1) | 679(58.4) | 761(64.5) | 1385(63.6) | 557(60.2) |
|  | Single | 2384(37.9) | 511(41.6) | 472(35.5) | 942(36.4) | 459(39.8) |
| Physical activity | |  |  |  |  |  |
|  | Never | 3620(60.1) | 717(58.6) | 712(55.9) | 1474(59.9) | 717(68.1) |
|  | Vigorous | 281(4.7) | 62(4.4) | 74(5.3) | 121(5.2) | 24(3.2) |
|  | Moderate | 1868(35.2) | 411(37.0) | 447(38.8) | 734(34.9) | 276(28.7) |
| Smoking status | |  |  |  |  |  |
|  | Never | 3237(56.1) | 779(65.6) | 744(61.2) | 1246(52.7) | 468(45.8) |
|  | Current | 1194(20.2) | 258(19.8) | 269(21.6) | 480(20.6) | 187(17.9) |
|  | Former | 1336(23.6) | 153(14.5) | 219(17.2) | 602(26.7) | 362(36.2) |
| Drinking status | |  |  |  |  |  |
|  | Never | 777(13.6) | 149(12.5) | 170(13.2) | 313(13.6) | 145(15.5) |
|  | Current | 3452(80.7) | 764(83.9) | 776(82.8) | 1397(80.1) | 515(74.6) |
|  | Former | 328(5.8) | 42(3.6) | 51(4.0) | 148(6.3) | 87(9.8) |
