# Supplementary material for: Trends and all-cause mortality associated with multimorbidity of non-communicable diseases among adults in the United States, 1999-2018: a retrospective cohort study
Source: Epidemiol Health. 2023 Feb 14;45:e2023023. doi: 10.4178/epih.e2023023 (PMC10586926; doi:10.4178/epih.e2023023)
Supplement: Supplementary Material 10. — eTable 9. Sample Size for Multimorbidity of NCDs among Adults in US by Sociodemographic, NHANES 2015-2016 (N(weighted %)) [file epih-45-e2023023-Supplementary-10.docx]

Supplementary Material 10: eTable 9. Sample Size for Multimorbidity of NCDs among Adults in US by Sociodemographic, NHANES 2015-2016 (N(weighted %))

|  |  |  | No. of Participants by Category of NCDs (Weighted %) | | | |
| --- | --- | --- | --- | --- | --- | --- |
|  | | Total | S[0] | S[1] | S[2~4] | s[5+] |
| Overall | | 5719(100.0) | 1151(20.7) | 1263(23.2) | 2275(39.7) | 1030(16.3) |
| Age | |  |  |  |  |  |
|  | 20~39 | 1953(36.2) | 753(66.0) | 632(51.3) | 534(25.1) | 34(3.8) |
|  | 40~64 | 2388(43.4) | 344(30.7) | 494(40.5) | 1094(49.6) | 456(48.5) |
|  | 65~ | 1378(20.4) | 54(3.2) | 137(8.2) | 647(25.2) | 540(47.6) |
| Sex | |  |  |  |  |  |
|  | Male | 2747(48.1) | 578(51.1) | 604(48.9) | 1070(47.0) | 495(45.7) |
|  | Female | 2972(51.9) | 573(48.9) | 659(51.1) | 1205(53.0) | 535(54.3) |
| Race /ethnicity | |  |  |  |  |  |
|  | Mexican American | 995(8.8) | 209(11.1) | 264(11.3) | 377(7.8) | 145(4.9) |
|  | Other Hispanic | 768(6.4) | 130(7.0) | 165(7.3) | 330(6.4) | 143(4.7) |
|  | Non-Hispanic White | 1863(63.9) | 311(57.1) | 382(61.8) | 730(65.2) | 440(72.4) |
|  | Non-Hispanic Black | 1198(11.3) | 210(11.2) | 244(10.6) | 528(12.2) | 216(10.6) |
|  | Other Race | 895(9.5) | 291(13.6) | 208(9.0) | 310(8.5) | 86(7.5) |
| Annual household income, $ | |  |  |  |  |  |
|  | <25000 | 1463(17.5) | 202(13.0) | 290(15.9) | 572(16.1) | 399(29.1) |
|  | 25000~75000 | 2395(43.2) | 508(43.9) | 550(43.0) | 939(41.6) | 398(46.7) |
|  | ≥75000 | 1434(39.3) | 333(43.1) | 343(41.1) | 607(42.3) | 151(24.2) |
| Educational attainment | |  |  |  |  |  |
|  | <High School | 1364(14.5) | 239(14.5) | 282(13.6) | 544(13.5) | 299(17.9) |
|  | High School | 1236(20.8) | 234(17.7) | 292(22.8) | 484(20.6) | 226(22.3) |
|  | >High School | 3114(64.8) | 677(67.8) | 689(63.6) | 1245(65.9) | 503(59.7) |
| Marriage Status | |  |  |  |  |  |
|  | Live together | 3441(63.9) | 682(60.4) | 785(65.1) | 1409(67.4) | 565(58.1) |
|  | Single | 2275(36.1) | 467(39.6) | 478(34.9) | 865(32.6) | 465(41.9) |
| Physical activity | |  |  |  |  |  |
|  | Never | 3352(51.4) | 687(52.4) | 698(48.1) | 1341(51.5) | 626(54.7) |
|  | Vigorous | 226(3.4) | 52(4.0) | 55(3.8) | 90(3.4) | 29(2.3) |
|  | Moderate | 2141(45.2) | 412(43.6) | 510(48.1) | 844(45.1) | 375(43.0) |
| Smoking status | |  |  |  |  |  |
|  | Never | 3321(56.4) | 791(67.6) | 796(61.2) | 1297(55.4) | 437(37.8) |
|  | Current | 1068(18.6) | 214(17.9) | 235(18.0) | 414(18.0) | 205(22.0) |
|  | Former | 1319(25.0) | 144(14.5) | 230(20.8) | 559(26.6) | 386(40.2) |
| Drinking status | |  |  |  |  |  |
|  | Never | 863(13.8) | 175(15.4) | 192(13.5) | 330(12.8) | 166(14.5) |
|  | Current | 3259(80.5) | 654(80.6) | 761(82.9) | 1331(80.7) | 513(76.1) |
|  | Former | 339(5.8) | 47(4.0) | 56(3.6) | 150(6.4) | 86(9.5) |
