# Supplementary material for: Trends and all-cause mortality associated with multimorbidity of non-communicable diseases among adults in the United States, 1999-2018: a retrospective cohort study
Source: Epidemiol Health. 2023 Feb 14;45:e2023023. doi: 10.4178/epih.e2023023 (PMC10586926; doi:10.4178/epih.e2023023)
Supplement: Supplementary Material 11. — eTable 10. Crude Weighted Trends in every NCD among total Adults in US, NHANES 1999-2018 (N(weighted %)) [file epih-45-e2023023-Supplementary-11.docx]

Supplementary Material 11: eTable 10. Crude Weighted Trends in every NCD among total Adults in US, NHANES 1999-2018 (N(weighted %))

| NCDs | Total | Trends in Multimorbidity of NCDs in NHANES Cycle Years | | | | | | | | | | Ratio of Prevalence: 2017–2018 vs 1999-2000 | *P*-trend |
| --- | --- | --- | --- | --- | --- | --- | --- | --- | --- | --- | --- | --- | --- |
|  | N=55081 | 1999-2000 | 2001-2002 | 2003-2004 | 2005-2006 | 2007-2008 | 2009-2010 | 2011-2012 | 2013-2014 | 2015-2016 | 2017-2018 |  |  |
| Diabetes | 8859(11.7) | 619(7.9) | 681(8.7) | 687(10.2) | 665(10.2) | 1054(12.4) | 1017(12.0) | 959(12.3) | 964(13.2) | 1039(13.5) | 1174(15.2) | 1.586 | <0.001 |
| Osteoporosis | 2598(5.2) | 179(3.3) | 329(5.0) | 375(6.0) | 254(5.0) | 395(5.5) | 348(4.9) | - | 322(5.3) | - | 396(6.1) | 1.503 | 0.008 |
| Obesity | 18688(33.4) | 1422(27.1) | 1432(27.1) | 1538(30.0) | 1647(32.6) | 2049(32.2) | 2285(34.8) | 1873(33.4) | 2083(36.4) | 2188(38.0) | 2171(40) | 1.400 | <0.001 |
| Liver condition | 2065(3.5) | 153(2.9) | 158(3.0) | 170(3.3) | 163(3.6) | 226(3.2) | 189(2.6) | 219(3.2) | 234(3.4) | 259(4.1) | 294(4.9) | 1.399 | <0.001 |
| Cancer | 5166(9.5) | 386(6.8) | 520(8.4) | 478(8.5) | 414(8.2) | 576(9.0) | 620(9.9) | 488(9.7) | 547(11.0) | 549(11.1) | 588(11.2) | 1.390 | <0.001 |
| Hyperlipidemia | 17857(31.4) | 1208(24) | 1372(23.9) | 1542(29.4) | 1418(29.8) | 1939(30.8) | 1904(29.2) | 2021(35.4) | 2193(37.5) | 2133(35.9) | 2127(35.3) | 1.373 | <0.001 |
| Weak/failing kidneys | 1611(2.5) | - | 145(1.9) | 144(2.1) | 136(2.2) | 179(2.4) | 158(1.7) | 200(2.8) | 187(2.6) | 239(3.4) | 223(3) | 1.333 | <0.001 |
| Thyroid problem | 4942(10.4) | - | 434(7.9) | 498(10.2) | 459(10.1) | 554(9.6) | 608(10.1) | 511(10.5) | 601(11.0) | 622(12.1) | 655(12) | 1.314 | <0.001 |
| Arthritis | 14692(24.4) | 1239(20.5) | 1331(20.7) | 1408(24.3) | 1247(24.3) | 1755(25.6) | 1674(23.8) | 1365(22.9) | 1508(26.5) | 1470(26.5) | 1695(27.9) | 1.266 | <0.001 |
| Stroke | 2197(2.8) | 190(2.3) | 204(2.4) | 212(2.7) | 193(2.9) | 258(3.2) | 227(2.6) | 229(2.8) | 202(2.9) | 209(2.7) | 273(3.3) | 1.256 | 0.025 |
| COPD | 679(3.7) | - | - | - | - | - | - | - | 192(3.4) | 194(3.1) | 293(4.5) | 1.181 | 0.152 |
| Gout | 1658(4.1) | - | - | - | - | 299(3.9) | 277(3.7) | 239(3.6) | 234(4.0) | 271(3.9) | 338(5.1) | 1.165 | 0.022 |
| Asthma | 7923(13.9) | 514(11.9) | 540(11.4) | 602(12.6) | 650(14.1) | 774(13.8) | 847(13.4) | 810(14.9) | 878(15.7) | 842(15.8) | 836(15) | 1.164 | <0.001 |
| Kidney stones | 3234(9.7) | - | - | - | - | 539(8.8) | 542(8.8) | 458(8.5) | 535(10.1) | 606(11.5) | 554(10.2) | 1.092 | 0.001 |
| Heart attack | 2465(3.4) | 221(3.3) | 258(3.2) | 274(3.9) | 215(3.5) | 282(3.3) | 261(3.1) | 203(3.2) | 230(3.4) | 251(3.3) | 270(3.7) | 1.067 | 0.907 |
| CHD | 2351(3.4) | 203(3) | 240(3.3) | 264(3.9) | 200(3.3) | 253(3.3) | 254(3.1) | 196(2.9) | 232(3.7) | 244(3.5) | 265(4.2) | 1.231 | 0.202 |
| CHF | 1906(2.4) | 167(2.1) | 187(2.2) | 197(2.6) | 180(2.6) | 217(2.4) | 174(2.0) | 187(2.7) | 182(2.6) | 214(2.5) | 201(2.3) | 1.052 | 0.499 |
| Hypertension | 21852(35.2) | 1783(29.5) | 1933(31.1) | 1988(34.8) | 1798(34.5) | 2396(34.8) | 2463(34.2) | 2250(36.1) | 2391(38.8) | 2353(37.4) | 2497(39.0) | 1.273 | <0.001 |
| Chronic bronchitis | 2292(5.9) | - | - | - | 290(6.6) | 375(5.9) | 311(4.7) | 297(5.8) | 320(5.7) | 304(5.7) | 395(6.7) | 1.013 | 0.662 |
| Emphysema | 1134(1.8) | 95(1.6) | 96(1.4) | 118(2.0) | 98(1.7) | 164(2.1) | 137(1.7) | 100(2.1) | 95(1.7) | 125(1.8) | 106(1.6) | 1.000 | 0.827 |
| Angina | 1624(2.4) | 180(2.8) | 199(2.9) | 212(3.1) | 155(2.5) | 164(2.1) | 155(1.9) | 129(2.3) | 136(2.2) | 133(2.0) | 161(2.6) | 0.954 | 0.019 |
