# Supplementary material for: Trends and all-cause mortality associated with multimorbidity of non-communicable diseases among adults in the United States, 1999-2018: a retrospective cohort study
Source: Epidemiol Health. 2023 Feb 14;45:e2023023. doi: 10.4178/epih.e2023023 (PMC10586926; doi:10.4178/epih.e2023023)
Supplement: Supplementary Material 12. — eTable 11. Crude Weighted Trends in every cancer among total Adults in US, NHANES 1999-2018 (N(weighted %)) [file epih-45-e2023023-Supplementary-12.docx]

Supplementary Material 12: eTable 11. Crude Weighted Trends in every cancer among total Adults in US, NHANES 1999-2018 (N(weighted %))

| NCDs | Total | Trends in Multimorbidity of NCDs in NHANES Cycle Years | | | | | | | | | | Ratio of  Prevalence:  2017–2018  vs 1999-2000 | P-trend |
| --- | --- | --- | --- | --- | --- | --- | --- | --- | --- | --- | --- | --- | --- |
|  | N=55081 | 1999-2000 | 2001-2002 | 2003-2004 | 2005-2006 | 2007-2008 | 2009-2010 | 2011-2012 | 2013-2014 | 2015-2016 | 2017-2018 |  |  |
| Testicular cancer | 31(0.2) | 3(<0.1) | 4(0.3) | 5(0.3) | 1(0.1) | 1(<0.1) | 3(0.1) | 5(0.3） | 3(0.2) | 4(0.5) | 2(0.4) | 6.189 | 0.147 |
| Other type of cancer | 349(0.7) | 21(0.3) | 41(0.7) | 28(0.5) | 24(0.5) | 25(0.4) | 40(0.6) | 34(0.9) | 39(0.7) | 43(0.8) | 54(1.1) | 2.165 | 0.001 |
| Non-melanoma skin cancer | 814(2.0) | 39(0.7) | 91(1.8) | 74(1.5) | 79(1.8) | 86(1.7) | 128(2.7) | 59(2.0) | 105(2.8) | 82(2.5) | 71(2.1) | 2.050 | <0.001 |
| Breast cancer | 813(2.9) | 63(1.9) | 72(2.5) | 67(2.3) | 77(3.0) | 93(2.8) | 95(3.1) | 66(2.0) | 99(3.5) | 84(3.0) | 97(4.4) | 1.737 | <0.001 |
| Kidney cancer | 108(0.1) | 6(0.1) | 13(0.1) | 14(0.2) | 5(0.1) | 7(0.1) | 12(0.2) | 10(0.1) | 12(0.1) | 8(0.2) | 21(0.2) | 1.700 | 0.128 |
| Thyroid cancer | 111(0.2) | 5(0.1) | 8(0.2) | 10(0.2) | 8(0.2) | 9(0.2) | 14(0.2) | 14(0.4) | 12(0.3) | 13(0.3) | 18(0.3) | 1.562 | 0.071 |
| Blood cancer | 13(<0.1) | 1(<0.1) | 1(<0.1) | - | 2(<0.1) | 1(<0.1) | 1(<0.1) | 1(<0.1) | 4(0.1) | 1(<0.1) | 1(<0.1) | 1.405 | 0.273 |
| Colon cancer | 396(0.5) | 32(0.4) | 41(0.5) | 39(0.4) | 24(0.3) | 51(0.7) | 53(0.7) | 40(0.6) | 28(0.6) | 36(0.4) | 52(0.7) | 1.385 | 0.043 |
| Brain cancer | 24(<0.1) | - | 3(<0.1) | 2(<0.1) | 2(<0.1) | 2(<0.1) | 4(<0.1) | 3(0.1) | 3(0.1) | - | 5(0.1) | 1.344 | 0.381 |
| larynx/windpipe cancer | 31(<0.1) | 4(<0.1) | 6(0.1) | 1(<0.1) | 2(<0.1) | 2(<0.1) | 4(0.1) | 6(<0.1) | 4(0.1) | - | 2(0.1) | 1.339 | 0.705 |
| Esophageal cancer | 32(0.1) | 2(<0.1) | 1(<0.1) | 4(0.1) | 2(<0.1) | 5(<0.1) | 2(<0.1) | 6(0.2) | 2(<0.1) | 2(<0.1) | 6(0.1) | 1.336 | 0.601 |
| Melanoma | 330(0.8) | 27(0.7) | 20(0.3) | 31(0.7) | 26(0.6) | 33(0.6) | 35(0.6) | 33(0.9) | 42(1.0) | 44(1.2) | 39(1.1) | 1.245 | 0.001 |
| Unknown skin cancer | 440(0.9) | 26(0.6) | 48(0.8) | 47(0.8) | 33(0.6) | 46(0.8) | 52(0.9) | 37(0.9) | 57(1.4) | 53(1.3) | 41(0.8) | 1.246 | 0.002 |
| Bladder cancer | 144(0.2) | 11(0.1) | 14(0.2) | 15(0.2) | 8(0.1) | 19(0.2) | 14(0.2) | 15(0.2) | 11(0.1) | 22(0.4) | 15(0.2) | 1.223 | 0.124 |
| Prostate cancer | 846(2.0) | 76(2.0) | 84(1.7) | 73(1.7) | 62(1.5) | 100(2.1) | 99(2.2) | 88(1.9) | 68(1.9) | 97(2.4) | 99(2.7) | 1.211 | 0.005 |
| Cervical cancer | 338(1.5) | 22(1.0) | 34(1.4) | 32(1.5) | 43(2.1) | 42(1.5) | 47(1.5) | 33(1.9) | 37(1.7) | 22(0.9) | 26(1.0) | 1.006 | 0.392 |
