# Supplementary material for: Trends and all-cause mortality associated with multimorbidity of non-communicable diseases among adults in the United States, 1999-2018: a retrospective cohort study
Source: Epidemiol Health. 2023 Feb 14;45:e2023023. doi: 10.4178/epih.e2023023 (PMC10586926; doi:10.4178/epih.e2023023)
Supplement: Supplementary Material 13. — eTable 11 continued. Crude Weighted Trends in every cancer among total Adults in US, NHANES 1999-2018 (N(weighted %)) [file epih-45-e2023023-Supplementary-13.docx]

Supplementary Material 13: eTable 11 continued. Crude Weighted Trends in every cancer among total Adults in US, NHANES 1999-2018 (N(weighted %))

| NCDs | Total | Trends in Multimorbidity of NCDs in NHANES Cycle Years | | | | | | | | | | Ratio of  Prevalence:  2017–2018  vs 1999-2000 | P-trend |
| --- | --- | --- | --- | --- | --- | --- | --- | --- | --- | --- | --- | --- | --- |
|  | N=55081 | 1999-2000 | 2001-2002 | 2003-2004 | 2005-2006 | 2007-2008 | 2009-2010 | 2011-2012 | 2013-2014 | 2015-2016 | 2017-2018 |  |  |
| Leukemia | 58(0.1) | 5(0.1) | 2(<0.1) | 8(0.1) | 1(<0.1) | 7(0.1) | 8(0.2) | 2(<0.1) | 5(0.1) | 12(0.4) | 7(0.1) | 0.989 | 0.03 |
| Lymphoma Hodgkin’s | 119(0.2) | 6(0.2) | 9(0.2) | 11(0.2) | 9(0.3) | 13(0.3) | 18(0.3) | 15(0.2) | 11(0.2) | 11(0.2) | 16(0.1) | 0.930 | 0.542 |
| Pancreatic cancer | 15(<0.1) | - | 1(<0.1) | - | - | 2(<0.1) | 1(<0.1) | 4(<0.1) | 3(<0.1) | 3(0.1) | 1(<0.1) | 0.926 | <0.001 |
| Rectal cancer | 35(0.1) | 6(0.1) | 6(0.1) | 2(<0.1) | 3(<0.1) | - | 4(0.1) | 5(0.1) | 1(<0.1) | 2(<0.1) | 6(0.1) | 0.921 | 0.600 |
| Uterine cancer | 244(0.8) | 23(0.9) | 28(0.9) | 25(0.7) | 19(0.7) | 23(0.6) | 32(0.8) | 22(0.8) | 18(0.5) | 32(1.2) | 22(0.8) | 0.892 | 0.985 |
| Mouth\tongue\lip cancer | 35(0.1) | 6(0.1) | 2(0.1) | 4(0.1) | 3(0.1) | 4(0.1) | 2(<0.1) | 1(<0.1) | 4(<0.1) | 4(0.2) | 5(0.1) | 0.870 | 0.693 |
| Stomach cancer | 43(<0.1) | 6(0.1) | 5(<0.1) | 3(<0.1) | 3(<0.1) | 6(0.1) | 3(<0.1) | 9(0.1) | 3(<0.1) | - | 5(<0.1) | 0.864 | 0.263 |
| Lung cancer | 152(0.2) | 13(0.3) | 22(0.3) | 15(0.3) | 11(0.2) | 17(0.3) | 6(0.1) | 14(0.2) | 21(0.4) | 15(0.2) | 18(0.2) | 0.784 | 0.443 |
| Ovarian cancer | 140(0.5) | 19(1.1) | 8(0.3) | 10(0.2) | 10(0.6) | 22(0.7) | 15(0.3) | 12(0.4) | 10(0.3) | 21(0.7) | 13(0.7) | 0.784 | 0.792 |
| Soft tissue cancer | 13(<0.1) | 2(<0.1) | 3(0.1) | 1(<0.1) | 1(<0.1) | 1(<0.1) | - | 1(<0.1) | 3(0.1) | - | 1(<0.1) | 0.748 | 0.571 |
| Liver cancer | 40(0.1) | 6(0.1) | 3(0.1) | 4(0.1) | 2(<0.1) | 4(<0.1) | 5(0.1) | 1(0.1) | 5(0.1) | 6(0.1) | 4(<0.1) | 0.687 | 0.92 |
| Bone cancer | 44(0.1) | 7(0.1) | 9(0.1) | 4(0.1) | 2(<0.1) | 5(0.1) | 4(<0.1) | 6(0.1) | 2(<0.1) | 2(<0.1) | 3(<0.1) | 0.634 | 0.091 |
| Nervous system cancer | 1(<0.1) | - | - | - | - | 1(<0.1) | - | - | - | - | - | - | - |
| Gallbladder cancer | 2(<0.1) | - | - | - | - | 1(<0.1) | - | - | - | - | 1(<0.1) | - | - |
