# Supplementary material for: Trends and all-cause mortality associated with multimorbidity of non-communicable diseases among adults in the United States, 1999-2018: a retrospective cohort study
Source: Epidemiol Health. 2023 Feb 14;45:e2023023. doi: 10.4178/epih.e2023023 (PMC10586926; doi:10.4178/epih.e2023023)
Supplement: Supplementary Material 14. — eTable 12. Weighted prevalence of every NCD in the age-subgroup and sex-subgroups, 2017-2018 (N(weighted %)) [file epih-45-e2023023-Supplementary-14.docx]

Supplementary Material 14: eTable 12. Weighted prevalence of every NCD in the age-subgroup and sex-subgroups, 2017-2018 (N(weighted %))

| NCDs | Age-subgroup | | | | |  | Sex-subgroup | | | |
| --- | --- | --- | --- | --- | --- | --- | --- | --- | --- | --- |
|  | 20~39 | 40~64 | 65~ | adj.F | P |  | Male | Female | adj.F | P |
| Asthma | 288(17.0) | 359(14.7) | 189(11.9) | 3.065 | 0.068 |  | 364(12.8) | 472(16.9) | 7.415 | 0.016 |
| Arthritis | 114(6.9) | 753(31.5) | 828(57.6) | 356.064 | <0.001 |  | 725(24.7) | 970(30.9) | 15.318 | 0.001 |
| CHF | 6(0.4) | 56(2.0) | 139(6.4) | 45.751 | <0.001 |  | 120(2.9) | 81(1.8) | 10.064 | 0.006 |
| CHD | 5(0.5) | 80(3.6) | 180(12.3) | 46.829 | <0.001 |  | 193(6.1) | 72(2.5) | 13.706 | 0.002 |
| Heart attack | 10(0.7) | 95(3.8) | 165(9.1) | 26.700 | <0.001 |  | 191(5.4) | 79(2.2) | 16.87 | 0.001 |
| Stroke | 8(0.2) | 104(3.0) | 161(9.5) | 72.952 | <0.001 |  | 136(2.9) | 137(3.8) | 2.66 | 0.124 |
| Emphysema | 2(0.1) | 44(2.2) | 60(3.1) | 19.438 | <0.001 |  | 66(1.7) | 40(1.5) | 0.272 | 0.610 |
| Thyroid problem | 83(5.3) | 265(12.2) | 307(23.5) | 96.000 | <0.001 |  | 164(4.8) | 491(18.7) | 73.912 | <0.001 |
| COPD | 6(0.3) | 123(5.7) | 164(9.4) | 49.602 | <0.001 |  | 155(4.1) | 138(4.8) | 0.846 | 0.372 |
| Gout | 14(0.9) | 144(6.1) | 180(10.4) | 36.642 | <0.001 |  | 232(7.1) | 106(3.2) | 26.064 | <0.001 |
| Hypertension | 277(17.6) | 1203(45.5) | 1017(63.2) | 225.076 | <0.001 |  | 1269(42.2) | 1228(36.1) | 11.793 | 0.004 |
| Hyperlipidemia | 191(11.7) | 974(41.3) | 962(64.3) | 238.483 | <0.001 |  | 1083(38.1) | 1044(32.7) | 11.701 | 0.004 |
| Diabetes | 71(3.8) | 553(17.4) | 550(30.7) | 145.959 | <0.001 |  | 608(16.0) | 566(14.4) | 0.923 | 0.352 |
| Obesity | 636(37.9) | 1003(43.1) | 532(37.0) | 3.442 | 0.047 |  | 988(40.1) | 1183(39.9) | 0.006 | 0.940 |
| Weak/failing kidneys | 23(1.4) | 74(2.6) | 126(6.6) | 16.738 | <0.001 |  | 116(3.1) | 107(2.9) | 0.144 | 0.710 |
| Kidney stones | 96(5.6) | 264(12.3) | 194(13.9) | 24.846 | <0.001 |  | 307(11.4) | 247(9.1) | 2.584 | 0.129 |
| Osteoporosis | - | 116(4.8) | 280(19.6) | 108.138 | <0.001 |  | 54(1.2) | 342(10.6) | 129.579 | <0.001 |
| Chronic bronchitis | 65(3.8) | 175(7.2) | 155(10.9) | 13.703 | <0.001 |  | 169(5.7) | 226(7.7) | 4.923 | 0.042 |
| Angina | 9(0.5) | 62(2.7) | 90(6.1) | 18.416 | <0.001 |  | 91(3.4) | 70(1.9) | 6.668 | 0.021 |
| Liver condition | 35(1.9) | 159(6.3) | 100(7.2) | 20.367 | <0.001 |  | 155(5.5) | 139(4.4) | 1.961 | 0.182 |
| Cancer | 34(2.6) | 188(10.0) | 366(28.8) | 125.171 | <0.001 |  | 276(9.7) | 312(12.5) | 7.508 | 0.015 |
